# Meeting Abstracts from the 77th Congress of the Italian Society of Pediatrics

**DOI:** 10.1186/s13052-023-01459-y

**Published:** 2023-07-07

## A1. Longitudinal Body Composition and anthropometric measurements in MIS-C children linked to Sars Cov-2 infection

### Marta Agostinelli^1^, Elisabetta Di Profio^1,2^, Martina C. Pascuzzi^1^, Sara Vizzuso^1^, Alessandro Leone ^3^, Elisabetta Bertoli^3,4^, Alessandra Bosetti^1^, Elvira Verduci^1,5^, Gian Vincenzo Zuccotti^1,6,7^

#### ^1^Department of Pediatrics, “Vittore Buzzi” Children’s Hospital, 20154 Milan, Italy; ^2^Department of Animal Sciences for Health Animal Production and Food Safety, University of Milan 20133 Milan, Italy; ^3^International Center for the Assessment of Nutritional Status (ICANS), Department of Food, Environmental and Nutritional Sciences, University of Milan, 20133 Milan, Italy; ^4^Istituto Auxologico Italiano, IRCCS, Lab of Nutrition and Obesity Research, Milan, Italy; ^5^Department of Health Sciences, University of Milan, 20142 Milan, Italy; ^6^Pediatric Clinical Research Center "Invernizzi", Department of Biomedical and Clinical Sciences "L.Sacco", University of Milan, 20157 Milan, Italy; ^7^Department of Biomedical and Clinical Science, “L. Sacco”, University of Milan, 20157 Milan, Italy

##### **Correspondence:** Marta Agostinelli

*Italian Journal of Pediatrics 2023*, **49(Suppl 1):**A1

**Background**

COVID-related Multi-Inflammatory Syndrome (MIS-C) is a rare condition observed in patients younger than 21 years of age with evidence of current or previous SARS CoV-2 infection, fever, elevated markers of inflammation and, at least, two signs of organ impairment [1]. A high body mass index (BMI) in patients with SARS CoV-2 infection was significantly correlated with the development of severe disease with the need for mechanical ventilation [2]. Changes in body composition in critically ill patients during long hospital stays are also described.

**Materials and methods**

Twenty-nine children and adolescents with MIS-C, defined according to CDC classification, were enrolled at the Vittore Buzzi Children's Hospital in Milan, between December 2020 and February 2021. Anthropometric parameters, including BMI and BMI z-score (according to CDC), waist and arm circumferences (AC) and triceps skinfolds (TFS), were measured for each patient at hospital admission, at discharge and after 10, 30, 90 and 180 days post discharge. Fat Mass (FM), Free Fat Mass (FFM) and Total Body Water (TBW) were detected by Electrical Bioimpedance at 10, 30, 90 and 180 days only in patients aged at least ≥7 years. Waist-to-height-ratio (WHR), A Body Shape Index (ABSI), Arm Muscle Area (AMA) and Arm Fat Area (AFA) were also calculated.

**Results**

The mean pre-hospitalization BMI z-score was 0.98 (0.54 to 1.43) and decreased to 0.66 (0.23 to 1.10) at recruitment. After discharge, BMI z-score increased by 0.33 (0.13 to 0.52) at 30 days and 0.25 (0.07 to 0.44) at 90 days. FM decreased 2.18 kg (0.2 to 4.16) at 30 days and 4.02 kg (2.13 to 5.92) at 180 days. In contrast, FFM increased by 1.29 kg (0.08 to 2.49) at 30 days and 4.31 kg (3.13 to 5.49) at 180 days. WHR decreased from 49.8 cm (47.7 to 52.0) to 48.2 cm (45.02 to 51.5) at 180 days. Compared with recruitment, AC, TSF, AMA, AFA increased by 1.43 cm (0.96 to 1.90), 2 mm (0.61 to 3.39), 2.17 cm2 (0.50 to 3.85) and 2.72 cm2 (1.44 to 4.00), respectively, at 180 days.

**Conclusions**

This observational study shows that during the acute phase of the disease the BMI z-score decreased and then returned to pre-hospital values within the first 30 days after discharge. An increase in anthropometric parameters of nutritional status (AC, AMA) and total FFM, and a decrease in total FM and WHR were also observed after discharge. These data suggest that children fully recovered their pre-admission nutritional status and body composition.

**Ethical approval**

The ethical approval was obtained from Institutional Review Board of the hospital (protocol Number 2021/ST/004). Children's parents gave their written consent for inclusion after being informed about the nature of the study.

**References**

1. CDC Health Alert Network. Multisystem Inflammatory Syndrome in Children (MIS-C) Associated with Coronavirus Disease 2019 (COVID-19). Available online: https://emergency.cdc.gov/han/2020/han00432.asp (accessed on January 2022).
2. Ong C, Lee J H, Senna S, Chia AZH, Wong JJM, Fortier MV, Leow MKS, Puthucheary ZA. Body composition and acquired functional impairment in survivors of pediatric critical illness. Crit Care Med. 2019; 47: 445–453.

## A2. Precocious Puberty: pandemic within pandemic

### Esther Angrisani^1^, Francesca Gaeta^1^, Elisabetta Placella^1^, Daniela Melis^2^, Carolina Mauro^3^

#### ^1^Scuola di Specializzazione in Pediatria, Dipartimento di Medicina, Chirurgia e Odontoiatria - Scuola Medica Salernitana, Università degli Studi di Salerno, Italia; ^2^ Professore Scuola di Specializzazione in Pediatria, Dipartimento di Medicina, Chirurgia e Odontoiatria - Scuola Medica Salernitana, Università degli Studi di Salerno, Italia; ^3^ U.O.C. Clinica Pediatrica. A.O.U. “San Giovanni di Dio e Ruggi d’Aragona”, Salerno, Italia

##### **Correspondence:** Esther Angrisani (es.angrisani@gmail.com)

*Italian Journal of Pediatrics 2023*, **49(Suppl 1):**A2

**Background**

Precocious puberty (PP) is the abnormal onset of pubertal development before the age of eight in females and nine in males. With an incidence of 1-5000/10000, it is considered one of the rare diseases.

During the lockdown there has been an increase in diagnoses of central PP (PPC); many factors are involved [1]: changing eating habits [2], sedentary lifestyle [2,3], increasing BMI [1,2,3], stress [2,3], increased use of electronic devices [1,2,3], and the consequent decrease in serum vitamin D levels [2] due to lack of sunlight exposure. The Italian data [3,4] are in agreement with the international ones^5^, to the point that it can be considered a 'pandemic within a pandemic'. The case study at hand is the clinical case of Martina, a seven-year-old girl referred by the treating paediatrician for premature pubarche.

In the anamnesis appeared hair, at first sparser, then thicker, on the patient's pubis, six months earlier, during lockdown. She visited the paediatrician at the age of seven years and ten months; at clinical evaluation: auxological parameters >97°ct and pubertal development B2 P2 A1. Given the unequivocal signs of pubertal activation, Martina was referred to paediatric endocrinology.

**Materials and methods**

When Martina came to our attention, we proceeded to anamnestic collection and clinical examination with auxological evaluation. Because PP was suspected, an X-ray of the wrist and non-dominant hand, pelvic ultrasonography with hysterometry and haematochemical tests for hormone assays were requested.

**Results**

Bone age, calculated according to the TW2 method, resulted increased by one year compared to the chronological age. Pelvic ultrasound shows uterus of prepubertal size and shape. After evaluation of gonadotropins and E2, GnRH-test for LH and FSH was performed, the result of which confirmed the activation of the hypothalamic-pituitary-gonadal axis. To complete the diagnosis, an encephalus MRI with gadolinium has been performed, which proved negative.

**Conclusions**

A diagnosis of PPC is made and therapy with a GnRH analogue is prescribed.

The first point to focus upon is the delay in diagnosis and, consequently, the late start of therapy: the spread of the SarsCov2 infection, the lockdown, the perception of clinics and hospitals as places of contagion, meant that the patient's first access was ten months after the onset of signs of pubertal activation.

The second point to be emphasised is the increase in PPC diagnoses, especially in the female sex, that occurred during the pandemic. Many triggers^1,2,3^ have been proposed and, although there is to date no known cause-effect relationship, it is imperative to research how, from a pathophysiological point of view, these factors are involved in the early onset of puberty.

**Consent to publish**

Written informed consent was obtained from the parents of the patient for publication of this case report and any accompanying images.

**References**

1. Stagi S, De Masi S, Bencini E, et al. Increased incidence of precocious and accelerated puberty in females during and after the Italian lockdown for the coronavirus 2019 (COVID-19) pandemic. Ital J Pediatr. 2020;46:165.
2. Street ME, Sartori C, Catellani C, Righi B. Precocious Puberty and Covid-19 Into Perspective: Potential Increased Frequency, Possible Causes, and a Potential Emergency to Be Addressed. Front Pediatr. 2021;9:734899.
3. Chioma L, Bizzarri C, Verzani M, et al. Sedentary lifestyle and precocious puberty in girls during the COVID-19 pandemic: an Italian experience. Endocr Connect. 2022;11:e210650.
4. Verzani M, Bizzarri C, Chioma L, Bottaro G, Pedicelli S, Cappa M. "Impact of COVID-19 pandemic lockdown on early onset of puberty: experience of an Italian tertiary center". Ital J Pediatr. 2021;47:52.
5. Acar S, Özkan B. Increased frequency of idiopathic central precocious puberty in girls during the COVID-19 pandemic: preliminary results of a tertiary center study. J Pediatr Endocrinol Metab. 2021;35:249-251.

## A3. The Transition in patients with Juvenile Idiopathic Arthritis: the experience in a pediatric centre and in an adult centre during the Covid-19 pandemic

### Francesca Ardenti Morini^1^, Francesca Soscia^1^, Fortunata Sabrina Civitelli^1^, Federica Ferrari^1^, Donatella Fiore^2^, Elisabetta Cortis^1^

#### ^1^UOC Pediatria, Ospedale Sant'Eugenio, Roma, Italia; ^2^UOS di Reumatologia, Nuovo Regina Margherita, Roma, Italia

##### **Correspondence:** Francesca Ardenti Morini (francesca.ardentimorini@aslroma2.it)

*Italian Journal of Pediatrics 2023*, **49(Suppl 1):**A3

**Background**

Young patients suffering from rheumatological chronic diseases face the difficult process of transition from the adolescence to the adulthood. This passage is known as “Transitional Care” or “Transition” [1]. The aim of the study is to describe the experience of transition of patients with rheumatological disease from a pediatric centre to an adult centre within two Hospitals in Rome.

**Materials and methods**

During the Covid-19 pandemic, a collaboration between the Reumathology Pediatric Centre of S.Eugenio and the Rheumatology Adult Centre of Ospedale Nuovo Regina Margherita has begun with the purpose of following the passage of the patients from one centre to another. By January 2021, 42 patients have been transitioned. Two clinical evaluations took place at Rheumatology Adult Centre in the presence of both professional figures - pediatric and adult rheumatologists.

Before each encounter, the clinical case was discussed through video conference. The first medical examination occurred in the presence of a caregiver and, during the second one, there was the possibility to choose or not the presence of one parent. The Transition is possible if the disease condition is stable or in remission.

**Results**

Throughout the pandemic period, from January 2021 to date, 42 patients between 15 and 18 years old have been transitioned (28 females and 14 males).

The two centres have shared information and therapeutic choices of each patient and there was no loss in therapeutic adherence nor any abandon from the adult centre.

**Conclusions**

The Transition of patients with chronic disease requires a comparison among rheumatology pediatric and adult colleagues in order to define an appropriate follow-up [2], to avoid the non therapeutic adherence and to ensure that the last one is adequately shared in the circumstance of the transition [3]. The collaboration model Pediatric Centre-Adulthood Centre allows the possibility of both examining and describing jointly complex clinical cases with enormous benefits for patients care.

**Ethical approval**

The study was approved by our Institution’s Ethics Board (Comitato Etico CE Lazio 2).

**Consent to publish**

Informed consent to publish has been obtained from these patients with a written consent signed by parents.

**References**

1. Ravelli A, Sinigaglia L, Cimaz R, Alessio M, Breda L, Cattalini M, et al. Transitional care of young people with juvenile idiopathic arthritis in Italy: results of a Delphi consensus survey. Clin Exp Rheumatol. 2019;37:1084-1091.
2. Lawson EF, Mellins ED. Paediatric rheumatic diseases: Navigating the transition from paediatric to adult care. Nat Rheumatol. 2017;13:138-9.
3. Titler G, Allen R. Rheumatology and the adolescent patient. Aust Fam Physician. 2017;46:558-63.

## A4. POCUS and ping-pong fractures: ultrasound as a valuable aid in cranial injuries

### Desirée Balconara^1^, Maria C. Finocchiaro^2^, Gianluca Trobia^2^ , Antonella V. Di Stefano^2^

#### ^1^ Postgraduate Training Program in Pediatrics, Department of Clinical and Experimental Medicine, University of Catania, Catania, Italy; ^2^U.O.C. Pediatric and Pediatric Emergency Room, Cannizzaro Emergency Hospital, Catania, Italy

##### **Correspondence:** Desirée Balconara (desireeb@live.it)

*Italian Journal of Pediatrics 2023*, **49(Suppl 1):**A4

**Background**

The peculiar malleability of the cranial bones of the newborn and infant predisposes to a depressed fracture defined as "ping-pong", due to the characteristic oval aspect assumed by the cranial vault, which recalls the deformation of a ping-pong ball. Characterized by the continuity of the cortex and by the prevalent involvement of the parietal bones, it can occur following a difficult birth or postnatal trauma; the underlying parenchymal involvement is usually minimal and rarely involves sequelae. Although Computed Tomography represents the diagnostic gold standard [1], point of care ultrasound (POCUS) is playing an increasingly decisive role in the early detection of skull fractures and their monitoring. We report the case of a child who was found to have a ping-pong fracture and who was monitored with the help of POCUS.

**Case report**

L.D. , 12 months, arrived at our Emergency Room for the finding of a right parietal depression after an accidental fall from a height of one meter. The general examination and neurological evaluation were normal. For the presence of an oval depression of about 3 cm in diameter, in the absence of other signs and symptoms, CT of the brain was required which showed right parietal fracture, without hemorrhagic or structural brain lesions (Figure 1). Bed-side ultrasound of the skull performed with a linear probe at a frequency between 7 and 10 MHz documented a depressed fracture of 0.32 cm (Figure 2). The neurosurgical evaluation did not suggest any intervention. By means of follow-up ultrasound checks, the fracture was gradually smoothed out (Figure 3).

**Conclusions**

The ping-pong fracture has an estimated incidence in Western countries between 4-10 thousand cases out of 100 thousand. Unlike the neonatal period where it recognizes age-related predisposing factors, in other periods, maltreatment should be considered, especially if there is involvement of other bone districts or any parenchymal suffering. Currently there are no shared protocols on the treatment of “ping pong" fractures and three possible approaches are recognized: wait-and-see, reduction with suction cup, neurosurgery [2]. Neurosurgeon determines the best option based on the characteristics of the fracture; generally, for depressions of less than 5 mm, "wait and see" is chosen, reserving the interventional options for depressions > 5 mm at potential risk of cerebral hypoperfusion. Although pending consensus on the timing of application and diagnostic accuracy of POCUS for fractures, updated literature reviews have reported sensitivity ranging from 88 to 100%, assigning this method a promising role in identification and non-invasive monitoring of traumatic cranial lesion.

**Consent to publish**

Informed consent to publish has been obtained from the patient's parents.

**References**

1. Dehbozorgi A, Mousavi-Roknabadi RS, Hosseini-Marvast SR, Sharifi M, Sadegh R, Farahmand F, Damghani F. Diagnosing skull fracture in children with closed head injury using point-of-care ultrasound vs. computed tomography scan. Eur J Pediatr. 2021;180:477-484.
2. Rizzello E, Migliarino V, Prisco A, Rabach I, Barbi E, Cozzi G. Due casi di frattura depressa del cranio o frattura “ping pong”. Medico e Bambino. 2022;25:e6-e9.

**Fig. 1 (abstract A4):**
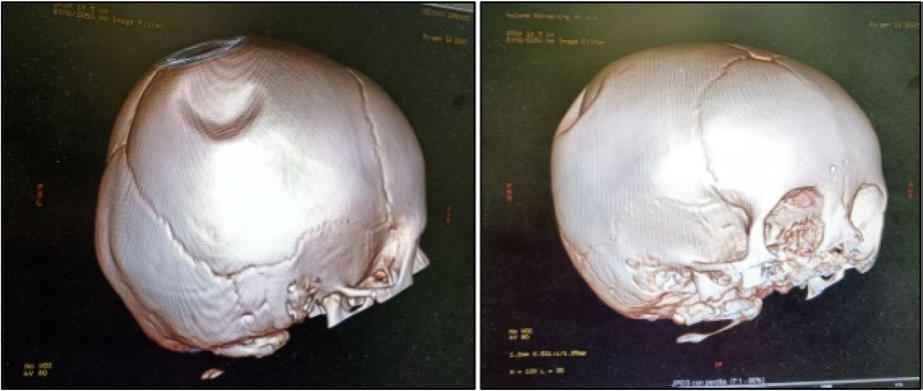
**.** Skull CT images with 3D reconstruction, performed a few hours after the trauma

**Fig. 2 (abstract A4):**
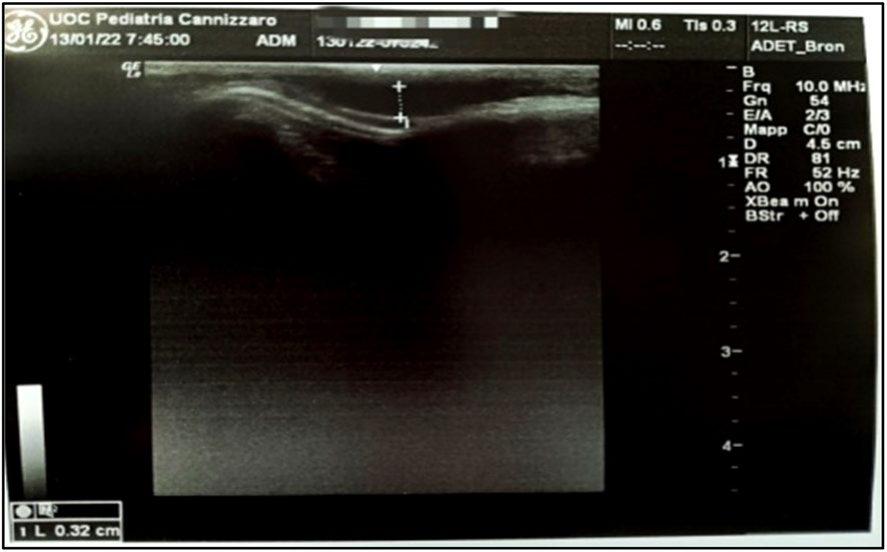
**.** POCUS image, performed at the same time as the CT

**Fig. 3 (abstract A4):**
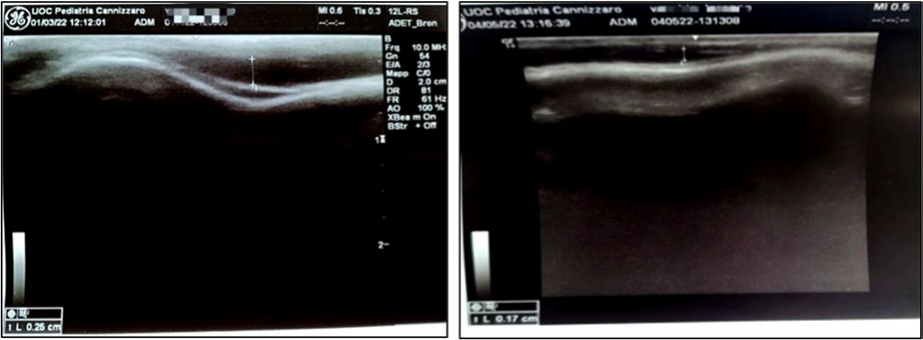
**.** a/b. POCUS image, taken 47 days (a) and 113 days (b) after the trauma

## A5. Verheij Syndrome: a case report of the first Italian patient with Chromosome 8q24.3 Deletion Syndrome

### Maria Serena Battigaglia, Valeria Tallarico, Giulia Pelaia, Lucy Castaldo, Mirella Petrisano, Elisabetta Muto, Simona Sestito, Daniela Concolino

#### Pediatric Unit, Magna Graecia University, Catanzaro, Italy

##### **Correspondence:** Maria Serena Battigaglia (mariaserena.battigaglia@gmail.com)

*Italian Journal of Pediatrics 2023*, **49(Suppl 1)**:A5

**Background**

Verheij syndrome was first described in 2009 [1] and just over 30 cases with this condition have been reported in the literature to date. It can be caused by the deletion of the 8q24.3 region or by the PUF60 gene mutation. The PUF60 gene is involved in embryological processes such as ocular plate closure and cervical spine segmentation [2]. The 8q24.3 region also contains the SCRIB gene, the elimination of which would cause microcephaly and stratification of the cortex [3]. Verheij Syndrome is characterized mainly by global developmental delay, intellectual disability, short stature. Other frequent features include: microcephaly, short neck, hand abnormalities, cardiac, ocular, urogenital, ear, skeletal abnormalities, joint laxity.

**Case presentation**

The patient, third child of unrelated parents, born at term from an uneventful pregnancy, came to our observation at the age of 7 years. In the first months of life, she showed: hypotonia, delayed psychomotor development and dysmorphisms, for which she carried out various specialist consultations, without obtaining an accurate diagnosis. On physical examination we observed: microcephaly, short neck, brachydactyly, short stature, ligament laxity, dentition abnormalities, speech difficulties. To the diagnostic investigations: asymmetry of the temporal horns on cerebral magnetic resonance, slowing of cerebral electrogenesis on electroencephalogram, dysmorphic appearance of the body of cervical vertebra C4 on x-ray, strabismus, patent Botallo's duct, inhomogeneous and smaller thyroid gland. For the presence of dysmorphic signs associated with developmental delay and organ malformations, she performed CGH-Array. A de novo 8q24.22q24.3 deletion has been detected, thus leading to diagnosis of Verheij syndrome. Compared to the other cases described in literature [2, 4, 5], our patient did not present uro-genital and otolaryngological abnormalities, while she showed thyroid and dentition anomalies, never previously described.

**Conclusions**

We have presented the case of a rare syndrome, difficult to diagnose both for the wide phenotypic spectrum and for the still limited knowledge. The careful analysis of the microdelete region allowed us to reach a diagnosis, to set up a correct follow-up. This case contributes to broaden the knowledge on a disease still poor known, and consequently rarely diagnosed.

Consent to publish

We obtained the written consent for publication from the guardian of the patient.

**References**

1. Verheij JB, de Munnik SA, Dijkhuizen T, de Leeuw N, Olde Weghuis D, van den Hoek GJ, Rijlaarsdam RS, Thomasse YE, Dikkers FG, Marcelis CL, van Ravenswaaij-Arts CM. An 8.35 Mb overlapping interstitial deletion of 8q24 in two patients with coloboma, congenital heart defect, limb abnormalities, psychomotor retardation and convulsions. Eur J Med Genet. 2009;52:353-7.
2. Yamada M, Uehara T, Suzuki H, Takenouchi T, Kosaki K. Protein elongation variant of PUF60: Milder phenotypic end of the Verheij syndrome. Am J Med Genet A. 2020 ;182:2709-2714.
3. Ezan J, Moreau MM, Mamo TM, Shimbo M, Decroo M, Richter M, Peyroutou R, Rachel R, Tissir F, de Anda FC, Sans N, Montcouquiol M. Early loss of Scribble affects cortical development, interhemispheric connectivity and psychomotor activity. Sci Rep. 2021;11:9106.
4. Xu Q, Li CY, Wang Y, Li HP, Wu BB, Jiang YH, Xu X. Role of PUF60 gene in Verheij syndrome: a case report of the first Chinese Han patient with a de novo pathogenic variant and review of the literature. BMC Med Genomics. 2018;11:92.
5. Graziano C, Gusson E, Severi G, Isidori F, Wischmeijer A, Brugnara M, Seri M, Rossi C. A de novo PUF60 mutation in a child with a syndromic form of coloboma and persistent fetal vasculature. Ophthalmic Genet. 2017;38:590-592.

## A6. Pediatric Acute Respiratory Distress Syndrome during COVID19 infection in two fragile patients

### Vittoria Berveglieri^1^, Valentina Folgheraiter^1^, Chiara Pavan^1^, Veronica Bertozzi^2^, Paolo Biban^3^

#### ^1^Pediatric Resident, University of Verona, Italy; ^2^ Attending Physician, Division of Pediatric Critical Care, Pediatric Intensive Care Unit, University Hospital of Verona, Italy; ^3^Director, Division of Pediatric Critical Care, Pediatric Intensive Care Unit, University Hospital of Verona, Italy

##### **Correspondence:** Vittoria Berveglieri

*Italian Journal of Pediatrics 2023*, **49(Suppl 1):**A6

**Background**

Pediatric Acute Respiratory Distress Syndrome (PARDS) has a relatively low incidence in patients affected by SARS COV2 infection (5%). However, it is responsible for 24% of admissions in Pediatric Intensive Care Unit (PICU) due to COVID19 infection. The most vulnerable patients are those affected by multisystem syndromes and severe comorbilities. We present two cases of PARDS due to SARS COV2 infection admitted to the PICU of Verona University Hospital.

**Materials and methods**

All patients diagnosed with PARDS during SARS COV2 infection and admitted to PICU from March 2021 to February 2022 were analysed. Two cases of PARDS were observed, one was a 3-year-old boy affected by Trisomy 21 without any other comorbilities (case A), the other was a 9-year-old girl affected by a chromosomopathy complicated with epileptic encephalopathy and severe scoliosis with mild chronic respiratory restrictive disease (case B).

**Results – Case A**

A patient affected by syncytial viral pneumonia, who was initially responsive to high flow nasal cannulas (HFNC) and who worsened due to overlapped SARS COV2 infection, was admitted to PICU for respiratory distress (PaO2/FiO2=53). During hospitalization (26 days) the patient was treated with invasive mechanical ventilation (IMV) (6/26), but he progressively deteriorated and developed hypoxic respiratory failure and pulmonary hypertension, therefore needing high-frequency oscillatory ventilation (HFOV) (5/26) and inhaled nitric oxide (4/26). An early introduction of neurally-adjusted ventilatory assist (NAVA) allowed weaning from ventilator support with a well tolerated switch to non-invasive ventilation (NIV). Due to improvement of clinical conditions, the patient was treated with HFNC until complete resolution of oxygen-dependency.

**Results – Case B**

The patient was admitted to PICU (54 days) for PARDS in COVID19-related pneumonia, after failure of HFNC treatment in the pediatric department. Due to severe hypoxemia (PaO2/FiO2 53) she needed IMV (15/54) and prone positioning. Patient’s comfort improved applying NAVA modality during both IMV and NIV. The clinical course was complicated by hemodynamic instability responsive to inotropic therapy. A slow improvement with prolonged oxygen-dependency was observed until she reached pre-hospitalization status.

**Conclusions**

Because of increased risk of respiratory complications during COVID19 infection, our experience underlines the need for a rigorous monitoring of all patients with comorbilities and/or multisystem syndromes. Moreover the early use of NAVA modality seems to improve patient's comfort and to lead to an easier switch to non-invasive respiratory support.

**Consent to publish**

The consent to the publication of the cases (minor patients) was collected in written form with the signature of the parent.

## A7. A case of intestinal pneumatosis in a 2-month-old-infant with SARS-COV-2 infection

### Giulia Biffi^1^ , Giada Maria Di Pietro^2^, Claudia Tagliabue^2^, Raffaella Pinzani^2^, Federica Giorgetti^1^, Arianna Petrillo^1^, Paola Giovanna Marchisio^1, 2^, Samantha Bosis^2^

#### ˡ Università degli Studi di Milano, Milano, Italy; ^2^ Fondazione IRCCS Ca’ Granda Ospedale Maggiore Policlinico, UOSD Pediatria Alta Intensità Cura, Milano, Italy

##### **Correspondence:** Giulia Biffi (giulia.biffi@unimi.it)

*Italian Journal of Pediatrics 2023*, **49(Suppl 1):**A7

**Background**

In most cases, SARS-CoV-2 infection in pediatric patients presents with fewer symptoms and is less severe than in adults. However, more severe forms have been reported in the literature, including cases of necrotizing enterocolitis (NEC) in premature infants with COVID19.

We describe a case of intestinal pneumatosis in an immunocompetent, two-month-old term infant, who was admitted to our hospital for feeding refusal and hematochezia for about 48 hours, in apyrexia.

**Case report**

Upon admission, a complete physical examination to evaluate the general clinical conditions and possible causes of evident hematochezia was performed. Blood tests including inflammation indexes, hemoglobin and an allergological panel (total Ig-E and specific Ig-E for milk and dairy products to exclude cow's milk proteins allergy) were performed. The patient also underwent an abdomen X-ray and an abdominal ultrasound to identify a possible intestinal aetiology. Microbiological tests were also performed including stool test for viruses and bacteria (antigenic tests and cultural examination) and polymerase-chain reaction (PCR) for SARS-CoV-2 both on nasopharyngeal swab and feces.

The physical examination revealed a healthy-appearing infant with unremarkable abdominal and perianal examination. The blood exams revealed a severe anemia with a hemoglobin of 6.9 g/dl requiring a blood transfusion. The abdominal ultrasound was negative while the abdomen X-ray showed an irregular appearance of the colon compatible with pneumatosis/NEC. The PCR for SARS-CoV-2 on nasopharyngeal swab and stool resulted positive. On the other hand, the further bacteriological and virological investigations on stool were negative. Allergological tests were positive and compatible with cow's milk proteins allergy. Intravenous antibiotic therapy with ceftriaxone (100 mg/kg/day tid) was administered for 7 days associated with intestinal rest. In the following days, clinical and radiological resolution of the pneumatosis was observed. Enteral feeding with hydrolyzed milk was then resumed, with subsequent adequate weight gain. Both the nasopharyngeal swab and stool test for SARS-CoV-2 detection were repeated after 7 days and resulted negative.

**Conclusions**

In most cases, COVID19 is asymptmatic or paucisymptomatic in infants and children. However, in some cases it could be associated with serious intestinal complications. Although in our case an allergy to cow's milk proteins was found, we can’t exclude that SARS-CoV-2 infection may have played a role in the pathogenesis or worsening of the intestinal disease.

**Consent to publish**

Informed consent to publish has been obtained from the parents of this patient.

## A8. Treatment of MISC with intravenous immunoglobuline-steroid association: personal case studies and comparison with literature

### Giulia M. Biondi^1^, Maria C. Finocchiaro^2^, Maria T. Garozzo^2^, Gian Luca Trobia^2^, Vita A. Di Stefano^2^

#### ^1^Department of Clinical and Experimental Medicine, Postgraduate Training Program in Pediatrics, University of Catania, Catania, Italy; ^2^Pediatric and Pediatric Emergency Room Unit, Cannizzaro Emergency Hospital, Catania, Italy

##### **Correspondence:** Giulia M. Biondi (giuliamarialidia@gmail.com)

*Italian Journal of Pediatrics 2023*, **49(Suppl 1):**A8

**Background**

Multisystem inflammatory disease (MISC) can occur 4-6 weeks after contact with SarsCov2. The clinical picture includes fever unresponsive to antipyretics associated with multiorgan dysfunction. Blood chemistry tests are characterized by signs of inflammation, coagulopathy and markers of organ damage [1].

From December 2020 to March 2022 at our OU we observed seven children aged between 4 and 13 with MISC.

**Materials and methods**

Our cases presented SarsCov2 positive serology, fever, marked asthenia, rash, conjunctival hyperaemia, elevated CRP, PCT, DDimer, hypoalbuminemia and hyponatremia. The most severe case showed cardiac involvement with increases in ferritin, transaminases, IL-6, troponin and ProBNP. All patients were closely monitored with the aid of POCUS and lung ultrasound. Among these, a 4-year-old boy showed hyper-refraction of the anterior descending coronary artery and pericardial effusion, a 6-year-old boy showed more altered ProBNP values with slight impairment of ventricular compliance, detachment of the pericardial sheets accompanied by slight pleural thickening. Sinus tachycardia and mild ventricular conduction delay were also noted in an 8-year-old boy. On the basis of both clinical and laboratory picture, immunoglobulins and intravenous steroids were administered with enoxaparin subcutaneously, applying guidelines developed by us in accordance with the most recent literature. In the four most serious patients, according to criteria for diagnosing Macrophage Activation Syndrome, it was necessary to use methylprednisolone as a 30 mg bolus for three days.

**Results**

Therapy with low-dose steroid-associated immunoglobulins allowed normalization of the temperature curve, haematochemical and ultrasound changes without relics in three out of seven cases. The failure of the first-line treatment with evolution to MAS, which occurred in four children, was successfully managed by administering a 30 mg bolus of intravenous corticosteroid.

**Conclusions**

MISC is a severe multisystem disease related to SARSCoV-2 infection. In the series presented, we used, as suggested by literature, immunoglobulins in association with intravenous methylprednisolone [2]. In consideration of cases of non-controlled evolution in MAS by immunoglobulins alone in association with intravenous low-dose methylprednisolone, close clinical-laboratory-radiological monitoring is imperative [3]. According to a review by Villacis-Nunez DS, published in March 2022, using steroids alone has shown promising results. In the aforementioned study, 215 patients with a mean age of 8 years were recruited and treated with three different therapeutic options: 69 patients with corticosteroids, 31 with IVIG and 115 with corticosteroid-associated IVIG. Patients treated with corticosteroids alone had milder disease at onset [4]. Intravenous steroid monotherapy in mild-course forms of MISC may be considered in future prospects.

The authors declare that they have no conflicts of interest.

**Consent to publish**

Written informed consent was obtained from parents for the processing of personal data and publication.

**References**

1. Reiff DD, Cron RQ. “Who would have predicted multisystem inflammatory syndrome in children?” Current rheumatology reports 2022;24:1-11.
2. Ouldali N, Toubiana J, Antona D, et al. Association of Intravenous Immunoglobulins Plus Methylprednisolone vs Immunoglobulins Alone With course of fever in multisystem inflammatory syndrome in children. JAMA. 2021;325:855–864.
3. Gowin E, Toczyłowski K, Sulik A, Wysocki J, Januszkiewicz-Lewandowska D. The role of glucocorticoids in the treatment of multisystem inflammatory syndrome (MIS-C)—Data from POLISH MIS-C Registry. Children 2022,9,178.
4. Villacis-Nunez DS, Jones K, Jabbar A, et al. Short-term outcomes of corticosteroid monotherapy in multisystem inflammatory syndrome in children. JAMA Pediatr. Published online March 28, 2022

## A9. Variability of clinical manifestations in paediatric COVID-19 infections

### Giusi M. Caltabiano^1^, Maria C. Finocchiaro^2^, Gianluca Trobia^2^, Antonella V. Di Stefano^2^

#### ^1^Postgraduate Training Program in Paediatrics, Department of Clinical and Experimental Medicine, Medical university, Catania, Italy; ^2^U.O.C. Paediatric and Paediatric Emergency Room, Cannizzaro Emergency Hospital, Catania, Italy

##### **Correspondence:** Giusi M. Caltabiano (gm.calta@outlook.it)

*Italian Journal of Pediatrics 2023*, **49(Suppl 1):**A9

**Background**

The variability of clinical manifestations in children with SARS-CoV-2 disease represents a challenge in emergency departments in terms of both early detection and targeted management [1]. The purpose of this monocentric retrospective observational study is to report the clinical characteristics of children with SARS-CoV-2 infection, evaluated at our paediatric emergency department from 8 March 2020 to 8 March 2022.

**Materials and methods**

Screening for COVID-19 was performed in dedicated areas, using rapid antigenic nasal or molecular nasopharyngeal swab in accordance with ministerial recommendations. Clinical and laboratory data were anonymised, retrieved from computerized electronic records and then collected through the GSA FINMATICA group. Microsoft Office Excel 2016 was used for statistical analysis.

**Results**

Out of a total of 11772 children evaluated to the emergency department in the time interval analysed, 121 patients were included, based on positive findings. The median age was 5.1 years (interquartile range: 0.02–14.03 years) (Table 1). Male sex accounted for 55% of cases. In 24% of cases the source of infection was known (school environment or family cluster). Fever was found in 73 patients (60%), followed by difficulty feeding and cough in 29% and 19%, respectively. Gastrointestinal symptoms were reported in 15% of cases. Neurological involvement was described in 9% of patients, with headache (6%) and seizures (3%). Accidental positive findings were recorded in 7% of patients affected by trauma. Three patients required ICU treatment: two, with comorbidities, due to status epilepticus and one infant with respiratory syncytial virus coinfection due to respiratory failure.

**Conclusions**

The children with SARS-CoV-2 infection who came to our observation in this two-year period presented, in accordance with the literature, nuanced and extremely variable clinical manifestations [2]. Hospitalization was necessary in 20% of the patients included in this study, half of them under the age of 1 year. Subjects admitted to intensive care were often affected by comorbidities. In order to contain the viral spread, it was necessary to reshape the organizational structure with the realization of separate paths to accommodate and manage the flow of patients.

**Consent to publish**

Informed consent to publish has been obtained from parents.

**References**

1. Nathan N, Prevost B, Sileo C, Richard N, Berdah L, Thouvenin G, Aubertin G, Lecarpentier T, Schnuriger A, Jegard J, Guellec I, Taytard J, Corvol H. The wide spectrum of COVID-19 clinical presentation in children. J Clin Med. 2020;9:2950.
2. Stacevičienė I, Burokienė S, Steponavičienė A, Vaičiūnienė D, Jankauskienė A. A cross-sectional study of screening for coronavirus disease 2019 (COVID-19) at the pediatric emergency department in Vilnius during the first wave of the pandemic. Eur J Pediatr. 2021;180:2137-2145.

**Table 1 (abstract A9):** **.** Characteristics of SARS-CoV-2 infected children

Parameter	Total (n)	Percentage (%)
Age (median)	5,1	-
Age groups		
<1	30	24.7
1-5	39	32.3
5-10	33	27.3
>10	19	15.7
Exposure history SARS-COV-2		
Known	29	23.9
Unknown	92	76.1
Sex		
Male	66	54.5
Female	55	45.5
Signs and symptoms		
Fever >37.5°C	73	60.3
Pharyngitis	35	28.9
Cough	23	19
Vomiting	11	9.1
Dyspnoea	9	7.4
Diarrhoea	7	5.7
Headache	7	5.7
Seizures	4	3.3
Others	8	6.6
Hospital admission		
Paediatric Unit	20	16.5
Intensive Care Unit	3	2.5

## A10. Culture screening with nasal and pharyngeal swab in patients with acute, chronic respiratory disease and with tracheostomy: a retrospective analysis

### Martina Carucci^1^, Martina Mazzoni^1^, Stefania Formicola^2^, Fabio Antonelli^2^, Eliana Brigante^2^, Barbara Borrelli^2^, Paolo Cavaliere^2^, Anna Naclerio^2^, Mariachiara Petagna^2^, Pierluigi Vuilleumier^2^

#### ^1^ Medicine, Surgery and Dentistry Department of “Scuola Medica Salernitana”, University of Salerno, Postgraduate School of Pediatrics, Baronissi, Italy; ^2^ Complex Operative Unit of Pneumology and UTSIR of the Santobono-Pausilipon Children’s Hospital, Naples, Italy

##### **Correspondence:** Martina Carucc (carucci.martina@gmail.com)

*Italian Journal of Pediatrics 2023*, **49(Suppl 1):**A10

**Background**

A potentially pathogenic polymicrobial flora is frequently found with culture screening in children with respiratory disease, especially if with chronic disease or with tracheostomy.

**Materials and methods**

The aim of the study is to evaluate the bacterial flora in the nasal swabs (NSs) and pharyngeal swabs (PSs) of patients admitted in the Complex Operative Unit of Pneumology and UTSIR of the Santobono-Pausilipon Children’s Hospital, from May 2021 to March 2022, categorized by comorbidity.

**Results**

99 of 203 patients had been admitted for acute respiratory disease, 73 of 203 for chronic respiratory disease, including 19 tracheostomized and 31 transferred from Pediatric Intensive Care Unit (PICU). 22.2% of all NSs and 32% of PSs tested positive for pathogenic microorganisms. Additionally, a polymicrobial flora was found in 18.9% and 35,2% of the above-mentioned percentages, respectively. Among the positive NSs, Staphylococcus Aureus (SA) was mostly found (48.9%) (Figure 1), followed by Pseudomonas Aeruginosa (PA) (15.6%); Klebsiella Pneumoniae (KP) (8.9%); Methicillin Resistant Staphylococcus Aureus (MRSA), Escherichia coli (EC) and Serratia Marcescens (SM) (4.4%); Enterococcus Faecium (EF), Enterobacter Aerogenes (EA), Stenotrophomonas Maltophilia (SM), Klebsiella Oxytoca (KO), Enterobacter Cloacae (ECL) and Candida Albicans (CA) (2.2%). 35.1% of patients with positive NSs had acute respiratory disease, 32,4% were transferred from PICU or/and were tracheostomized, 8.1% had acute-on-chronic respiratory disease. A polymicrobial flora was found in 0, 42,9 and 14,3% of the above-mentioned categories, respectively. Among the positive PSs, SA and PA were mostly found (22.8%) (Figure 2), followed by KP (13.9%); CA (10.1%); EC (8.9%); MS, Proteus Mirabilis, EA and MS (3.8%); MRSA (2.5%); KO, ECL and Haemophilus Influenzae (1.3%). 37% of patients with positive PSs had acute respiratory disease, 22.2% were transferred from PICU and/or were tracheostomized, 40.6% had acute-on-chronic respiratory disease. A polymicrobial flora was found in 15.8, 15.8 and 68.5% of the above-mentioned categories, respectively. Regarding the tracheostomized, 53.1% of NSs tested positive for SA (35.3%); PA and Enterobacter (11.8%); KB, CA, SM, MRSA, EC, EF, SM (5.9%). 64.7% of PSs tested positive for PA (22.7%); SA and KP (18.2%); CA (13.6%); EC (9.1%); SM, MRSA, Enterobacter (4.5%).

**Conclusions**

For each category of patients Staphylococcus Aureus is the most common microorganism, followed by Pseudomonas Aeruginosa and Klebsiella Pneumoniae, whereas the Methicillin Resistant Staphylococcus Aureus was poorly isolated. Patients with chronic disease, with tracheostomy or transferred from PICU, are more likely to have positive NSs and/or PSs and to be colonized by polymicrobial flora.

## A11. Humoral response to COVID-19 mRNA vaccines in a cohort of young kidney transplant recipients from a single Center in Northern Italy

### Marco Cazzaniga^1^, Olga Caporale^2^, Sara Testa^2^, Marta Brambilla^3^, Jessica Serafinelli^2^, Maria Viganoni^1^,Chiara Tamburello^2^ , Giovanni Montini^4^

#### ^1^ University of Milan. Fondazione IRCCS Ca' Granda Ospedale Maggiore Policlinico - Pediatric Nephrology- Dialysis and Transplantation Unit, Milan, Italy; ^2^ Fondazione IRCCS Ca' Granda Ospedale Maggiore Policlinico -Pediatric Nephrology- Dialysis and Transplantation Unit, Milan, Italy; ^3^ ASST Fatebenefratelli Sacco, Pediatric Unit, Milan, Italy; ^4^ Fondazione IRCCS Ca' Granda Ospedale Maggiore Policlinico - Pediatric Nephrology- Dialysis and Transplantation Unit, Milan, Italy. University of Milan, Department of Clinical Sciences and Community Health, Milan, Italy

##### **Correspondence:** Sara Testa (sara.testa@policlinico.mi.it)

*Italian Journal of Pediatrics 2023*, **49(Suppl 1):**A11

**Background**

Adult kidney transplant (KT) patients showed an impaired response to SARS-CoV-2 vaccine. We investigate immune-response to COVID-19 vaccines in young KT recipients from Northern Italy.

**Materials and methods**

We prospectively studied KT patients aged 12-25 years, managed in our Center on maintenance IS therapy (corticosteroids, CNI and anti-proliferative agents), eligible for antiSARS-Cov2 vaccination according to the schedule of the Italian Medicines Agency for immunosuppressed patients (two doses plus additional dose one month later). From 1st July 2021 to 28th February 2022 we evaluated antiSpike-protein antibody response at T0 (before vaccine), T1, T2 and T3 (14±3 days after 2nd and 3rd dose and 90±7 days after 3rd dose, respectively) to BNT162b2 (Pfizer/BioNTech) or mRNA-1273 (Moderna). AntiSpike total Ig titer cut-off was 0.8 U/ml (Roche® Elecsys Anti-SARS-CoV-2-S). Exclusion criteria: KT or additional IS within 6 months, relapse of primary disease, vaccine before KT, ongoing COVID-19, patients resident outside the Region.

**Results**

Eighty-seven patients were eligible; 68 patients were enrolled. Median age: 19.5 (IQR:16.3-21.9) years; median time from KT: 61.4 (IQR: 36.7-111.7) months. Five patients dropped out of study after enrollment.

Anti-SARS-Cov2 Spike Antibodies response to mRNA vaccines is shown in Figure 1; 90% of non-responders at T1 (20 patients) seroconverted at T3.

We didn’t find correlation between time from KT (the shorter time, the most intensive immunosuppression) and Ig-titer. Twelve out of 58 pts developed COVID19 after the third additional vaccine dose; in this population AntiSpike Ig titer at T2 was lower compared to the value of non infected patients, even if not statistically significant: 144 U/ml (IQR:9.4-3683) vs. 4771 U/ml (IQR:79.1-13000) respectively. None patient had side effects, including acute rejection episodes or de novo DSA development.

**Conclusions**

KT pediatric recipients exhibit a satisfactory response after 2 doses of vaccine, that become comparable to that of immunocompetent population after the third. Furthermore, the response after two doses is better if compared with adult KT population (63.6% vs 4-48% ) [1,2].

**Ethics Approval**

The local Institutional Review Board approved the study. Written informed consent was obtained from a parent or guardian or the participants themselves.

**References**

1. Benotmane I, Gautier-Vargas G, Cognard N, et al. Low immunization rates among kidney transplant recipients who received 2 doses of the mRNA-1273 SARS-CoV-2 vaccine. Kidney Int. 2021;99:1498-1500.
2. Rozen-Zvi B, Yahav D, Agur T, Zingerman B, Ben-Zvi H, Atamna A, et al. Antibody response to SARS-CoV-2 mRNA vaccine among kidney transplant recipients: a prospective cohort study. Clin Microbiol Infect. 2021;27:1173.e1-1173.e4.

**Fig. 1 (abstract A11):**
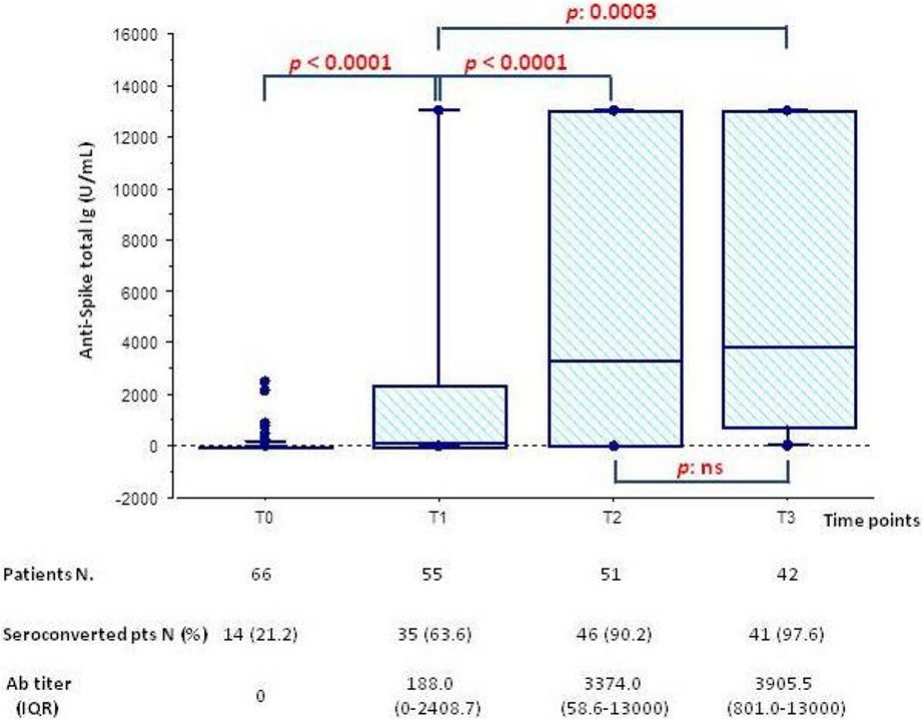
**.** Anti-SARS-CoV-2 Spike Antibodies response to mRNA vaccines

## A12. Association between symptoms and viral load in saliva specimens in a cohort of SARS-CoV-2-infected pediatric patients

### Benedetta Ciacchini^1^, Cinzia Borgogna^2^, Alice Monzani^1^, Chiara Grisaffi^1^, Michela Licitra^1^, Miriam Chiara Gatto^1^, Daniela Ferrante^2^, Enrico Felici^3^, Marisa Gariglio ^2^, and Ivana Rabbone^1^

#### ^1^Division of Pediatrics, Department of Health Science, University of Piemonte Orientale, Novara – Italy; ^2^ Division of Virology, Department of Translational Medicine, University of Piemonte Orientale, Novara – Italy; ^3^ Pediatric and Pediatric Emergency Unit, The Children Hospital, AO SS Antonio e Biagio e C.Arrigo, Alessandria - Italy

##### **Correspondence:** Benedetta Ciacchini

*Italian Journal of Pediatrics 2023*, **49(Suppl 1):**A12

**Background**

SARS-CoV2-infected children usually display mild symptoms or are asymptomatic. In the literature, there is no consensus on the association between symptoms and viral load in nasopharyngeal or saliva specimens. The gold standard for the diagnosis of the infection is quantitative polymerase chain reaction (qPCR) in nasopharyngeal swab (NPS) samples, but quantification of the viral load in saliva, which is less traumatic and easier to obtain, is a possible alternative.

**Materials and methods**

From November 2020 to May 2021, SARS-CoV-2 viral load was assessed by droplet digital PCR (ddPCR) in saliva specimens from non-vaccinated 35 pediatric patients who had a SARS-CoV2-positive nasopharyngeal swab (NPS). Saliva viral loads (SLV) were correlated with the cycle threshold (C_T_) values of qPCR on NPS and clinical symptoms.

**Results**

The median SARS-CoV-2 SVL was 0.56 copies/μl (range 0-344,000) and it was inversely correlated with the C_T_ values on NPS (R = -0.87, p <0.0001) thus revealing good consistency between the two methods. The prevalence of patients with a high SVL (> 100 copies) was 57% among children with nasopharyngeal symptoms, 40% with cough, 32% with fever, 33% with gastrointestinal symptoms and 17% with dyspnea. None of the children with headache, rash, conjunctivitis, or asymptomatic showed a high SVL (Fig. 1). Overall, the viral load was higher in patients with symptoms such as rhinitis and pharyngodynia compared to those who did not display these symptoms (p = 0.033, OR = 7.3), (Fig. 1).

**Conclusions**

Saliva can be considered an adequate sample for quantifying SARS-CoV-2 viral load. The SVL was high in more than half of the patients with nasopharyngeal symptoms, but in none of the asymptomatic patients or in those with headache, rash, conjunctivitis. Despite the limitation of low sample size, these data could be useful to better understand the dynamics of viral spreading throughout the pediatric population.

**Ethical Approval**

The study was approved by the Ethics Board of AOU “Maggiore della Carità” of Novara, approval number 008/21.

**Fig. 1 (abstract A12):**
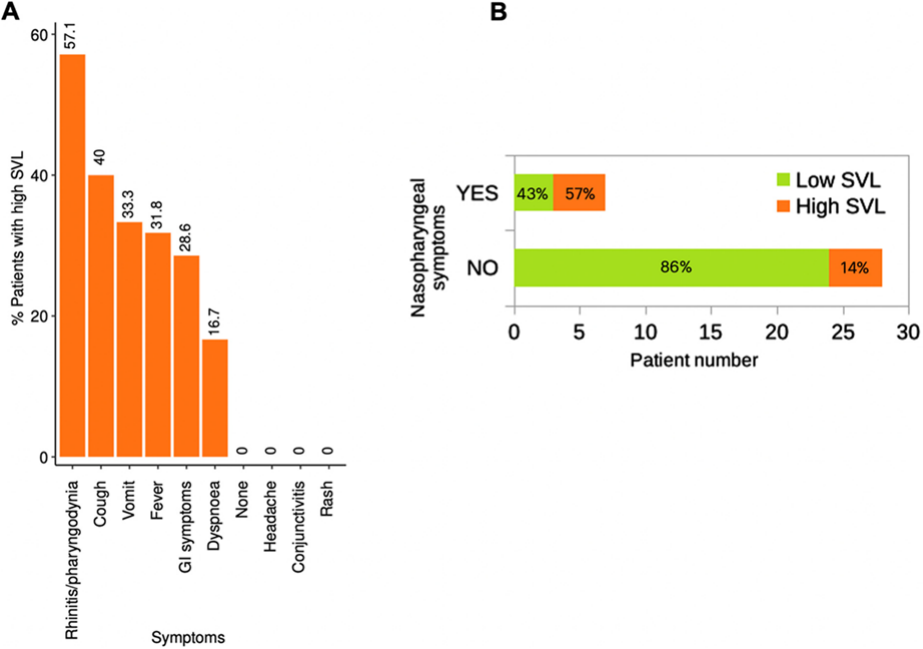
**.** Frequency of high SVL across different symptoms (A) and proportion of high SVL in patients with and without nasopharyngeal symptoms (B)

## A13. “L’ABCDEFG” of pediatric bedside lung ultrasound: an Italian protocol containing cornerstones of lung ultrasound semeiotics

### Laura Cozzi^1^, Giulia Biffi^1^, Alessia Rocchi^2^, Francesca Chiaraviglio^3^, Francesca Patria^4^, Paola Giovanna Marchisio^4,5^, Carlo Virginio Agostoni^6,7^

#### ^1^University of Milan, Milan, Italy; ^2^Pediatric Emergency Department, Fondazione IRCCS Ca' Granda Ospedale Maggiore Policlinico, Milan, Italy; ^3^Pediatric Radiology Unit, Fondazione IRCCS Ca' Granda Ospedale Maggiore Policlinico, Milan, Italy; ^4^Pediatric Highly Intensive Care Unit, Fondazione IRCCS Ca' Granda Ospedale Maggiore Policlinico, Milan, Italy; ^5^Department of Pathophysiology and Transplantation, University of Milan, Milan, Italy; ^6^Department of Clinical Sciences and Community Health, University of Milan, Milan, Italy; ^7^Pediatric Intermediate Care Unit, Fondazione IRCCS Ca' Granda Ospedale Maggiore Policlinico, Milan, Italy

##### **Correspondence:** Laura Cozzi (laura.cozzi@unimi.it)

*Italian Journal of Pediatrics 2023*, **49(Suppl 1):**A13

**Background**

Lung ultrasound has an increasingly important role in helping clinical examination, as a possible support tool for diagnostic decisions and therapeutic choices, and to define prognostic scores [1, 2]. This exam provides quick, bedside, and inexpensive information that is useful for acute and chronic patients [3]. The primary outcome of our study was to create a simplified protocol [4] based on the mnemonic acronym “L'ABCDEFG”, containing the cornerstones of lung ultrasound semeiotics. The secondary outcome was the implementation of recognition capabilities of ultrasound patterns, increasing inter-operator agreement, and the consequent use of ultrasound data in the management of clinical cases (following the protocol procedure).

**Materials and methods**

A bibliographic search was carried out (on PubMed), typing the keywords "pediatric" AND "lung" AND "ultrasound" and 50 studies published in the last 10 years were considered [1, 2, 3, 4]. Eight elements have been identified and considered "cornerstones" of lung ultrasound semeiotics. A written protocol was formulated. A lesson (of one hour) was offered, held by pediatric residents to other pediatric residents to illustrate it. Before and after the sharing of the protocol, a 21-question test was proposed to evaluate the correct interpretation and management of clinical cases assisted by lung ultrasound findings (images and videos). Finally, a statistical analysis of the data was performed.

**Results**

The "ABCDEFG" lung ultrasound protocol is defined as follows:

- **L** '- lung sliding, pleural line
- **A** - A lines
- **B** - B lines
- **C** - consolidation?
- **D** - dynamic bronchogram? (static? fluid?)
- **E** - B lines extension? (white lung?)
- **F** - fluid? (pleural effusion, curtain sign?)
- **G** - gap? (pneumothorax, pulmonary sign?)

53 pediatric residents participated and 50 completed the tests. Following the protocol, the percentage of correct answers increased by an average of 30%: correct answers were given on average in 54% of the pre-course test, compared to a result of 84% in the post-course test (Figure 1.). There was a greater inter-operator agreement in the post-course test both in the interpretation of lung ultrasound images and in their use as a diagnostic support tool.

**Conclusions**

The “ABCDEFG” protocol is a valid tool useful in performing lung ultrasounds. The ability to recognize lung ultrasound patterns and to answer clinical problems was notably better following the protocol. Bedside lung ultrasound is an area of great interest that should be taught during graduate school.

**Trial registration**

No trial registration

**Acknowledgements**

We thank pediatric residents of Clinica De Marchi for their participation.

**Consent to publish**

All participants consented to publication. The text was anonymous, pre-post exams were linked with a code number.

**Conflict of Interest**

The authors declare that the research was conducted in the absence of any commercial or financial relationships that could be construed as a potential conflict of interest.

**Ethics Approval**

No ethics approval was needed. No human material o animals was involved. All participants consented to the exam, which was anonymous. A privacy statement was signed before the exam. No individual pieces of information are published. Informed consent to publish has been obtained from participants. No conflict of interest.

**References**

1. Musolino AM, Tomà P, De Rose C, Pitaro E, Boccuzzi E, De Santis R et al. Ten Years of Pediatric Lung Ultrasound: A Narrative Review. Front Physiol. 2022;12:721951.
2. Rea G, Sperandeo M, Di Serafino M, Vallone G, Tomà P. Neonatal and pediatric thoracic ultrasonography. J Ultrasound. 2019;22:121-130.
3. Lovrenski J. Pediatric lung ultrasound - pros and potentials. Pediatr Radiol. 2020;50:306-313.
4. Zanforlin A, Smargiassi A, Perrone T, Inchingolo R, Torri E, Limoli G et al. Artifacts and Signs in Lung Ultrasound: The Need for a Revised Classification: Part 1: An Accademia di Ecografia Toracica (AdET) Survey: Part 1: An Accademia di Ecografia Toracica (AdET) Survey. J Ultrasound Med. 2022;41:2907-2909.

**Fig. 1 (abstract A13):**
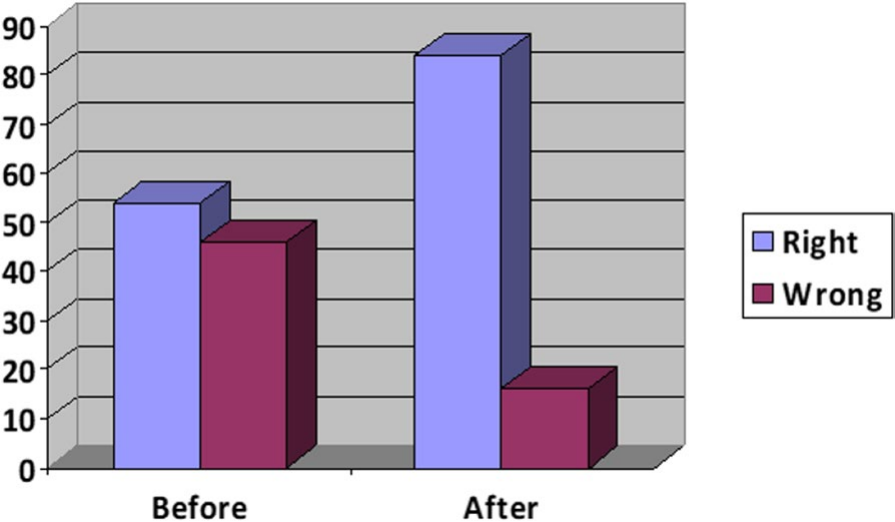
**.** The right answers increased by 30% after the course

## A14. Multisystem Inflammatory Syndrome COVID related: a South Italy Center’s experience

### Federica D’Amico, Cecilia Lugarà, Simone Foti Randazzese, Fabio Toscano, Pietro Basile, Eleonora Maria Patané, Arianna Torre, Giuseppina Zirilli, Giuseppina Salzano, Francesco Letterio De Luca, Giovanni Battista Pajno, Mariella Valenzise

#### Department of Human Pathology of Adulthood and Childhood, University of Messina, Messina, Italy

##### **Correspondence:** Federica D’Amico (federicadamico93@gmail.com)

*Italian Journal of Pediatrics 2023*, **49(Suppl 1):**A14

**Background**

Although the Sars-Cov2 infection has a milder course in the pediatric age [1], a small part of patients can develop much feared complications. One of them is the Multisystem Inflammatory Syndrome COVIDrelated (MIS-C) and its important impact on the public health [2].

**Materials and methods**

Between January 2021 and February 2022, 8 patients, 5 males (55%) and 3 females (35%), were hospitalized in our Pediatric Clinic with a MIS-C diagnosis. The inclusion criteria were: need for hospitalization, patients under the age of 18, fever, recent Sars-Cov2 infection and/or positivity for anti-Nucleocapsid antibodies. The diagnosis was foundend on laboratory (hemochrome, CRP, Troponin, proBNP, Ferritin, Triglycerides, Fibrinogen, D-Dimer) and instrumental exams (ECG, echocardiography, abdomen ultrasound, chest X-ray, brain CT, cardiac MRI) according to national and internationals consensus [3,4,5].

**Results**

The mean age was 8,2 ± 4,66 SDS. 6 patients (75%) had a recent infection due to Sars-Cov2 documented by nasopharyngeal swab. At the time of the hospitalization, 4 patients (50%) had abdominal pain, 1 (13%) dyspnea, 4 (50%) vomit, 3 (38%) diarrhea, 4 (50%) skin rash, 4 (50%) conjunctival hyperemia, 1 (13%) mucocutaneous lesions, 1 (13%) nuchal stiffness. The symptoms got worse after 7,75 ± 9,36 days. During the hospitalization, 8 patients (100%) had cardiological (63% reduction of ejection fraction, 38% cardiac effusion, 38% valvular insufficiency, 50% sinus tachycardia, 25% sinus bradycardia, 38% myocarditis), 6 (75%) abdominal (17% non-specific ileitis, 17% acute abdomen, 17% pancreatitis, 67% non-specific abdominal pain) and 3 (38%) respiratory complications (100% parenchymal thickening). At the laboratory assessment, the patients had the following values: White Blood Cells 8816,25 ± 4945,80 mmc, Lymphocytes 972,75 ± 276,08 mmc, pro-BNP 9171,13 ± 9516,35 pg/ml, Troponin T 113,1 ± 151,89 ng/L, Fibrinogen 559,25 ± 143,25 mg/dl, D-Dimer 2,25 ± 0,89 ug/dl. In all patients, the indices of inflammation were increased with mean values of 13,3 ± 8,8 mg/dl for CRP and 2451,08 ng/ml for Ferritin (Table 1). The therapeutic treatment consisted in: methylprednisolone boluses (88%), intravenous immunoglobulins (100%), Interleukin 1 receptor antagonist (25%), intensive care (50%). One patient had a Macrophage Activation Syndrome.

**Conclusions**

At the beginning, the most frequent symptoms were abdominal ones, followed by skin and respiratory manifestations. All patients presented cardiological involvement with laboratory, ECG and/or echocardiographic manifestations. The abdominal involvement mimicked pathologies of gastroenterological or surgical relevance [6,7]. All patients presented a quick clinical and laboratory response to the therapies, although the necessity of a long follow-up especially for the cardiological complications.

**Consent to publish**

Data were collected retrospectively on hospitalized patients after reading and signing of the informed consent by the parents. All data were collected ensuring patient anonymity. There aren’t data which allow to know patient’s identity.

**References**

1. Wu Z, McGoogan JM. Characteristics of and Important Lessons From the Coronavirus Disease 2019 (COVID-19) Outbreak in China: Summary of a Report of 72 314 Cases From the Chinese Center for Disease Control and Prevention. JAMA. 2020;323:1239–1242.
2. Levin M. Childhood Multisystem Inflammatory Syndrome - A New Challenge in the Pandemic. N Engl J Med. 2020;383:393-395.
3. Henderson LA, Canna SW, Friedman KG et al. American College of Rheumatology Clinical Guidance for Multisystem Inflammatory Syndrome in children associated with SARS-CoV-2 and hyperinflammation in pediatric COVID-19: Version 2. Arthritis Rheumatol. 2021;73:e13-e29.
4. T. Radia, N. Williams, P. Agrawal et al., Multi-system inflammatory syndrome in children & adolescents (MIS-C): A systematic review of clinical features and presentation, Paediatric Respiratory Reviews, https://doi.org/10.1016/j.prrv.2020.08.001.
5. Feldstein LR, Rose EB, Horwitz SM et al. Multisystem Inflammatory Syndrome in U.S. children and adolescents. N Engl J Med. 2020;383:334-346.
6. Sahn B, Eze OP, Edelman MC, Chougar CE, Thomas RM, Schleien CL, Weinstein T. Features of intestinal disease associated with COVID-Related Multisystem Inflammatory Syndrome in children. J Pediatr Gastroenterol Nutr. 2021;72:384-387.
7. Yock-Corrales A, Lenzi J, Ulloa-Gutiérrez R et al. Acute abdomen and appendicitis in 1010 Pediatric Patients With COVID-19 or MIS-C: A multinational experience from Latin America. Pediatr Infect Dis J. 2021;40:e364-e369.

**Table 1 (abstract A14):** **.** Clinical and laboratory assessment at the hospitalization

VARIABLES	AVERAGE	STANDARD DEVIATION
Worsening interval (days)	7,75	9,36
White Blood Cells (mmc)	8816,25	4945,8
Lymphocytes (mmc)	972,75	276,08
CRP (mg/dl)	13,3	8,8
Ferritin (ng/ml)	1228,25	2451,08
Pro-BNP (pg/ml)	9171,13	9516,35
Troponin-T (ng/ml)	113,1	151,89
Fibrinogen (mg/dl)	559,25	143,25
D-Dimer (μg/ml)	2,25	0,89

## A15. Twenty-year experience of food-induced anaphylaxis in children: A cluster analysis

### Maria De Filippo^1^,Martina Votto^1^, Riccardo Castagnoli^1^, Salvatore Fasola^2^,Stefania La Grutta^2^, Gian Luigi Marseglia^1^, Alessia Marseglia^1^, Amelia Licari^1^

#### ^1^Pediatric Unit, Department of Clinical, Surgical, Diagnostic, and Pediatric Sciences, University of Pavia, Pavia, Italy; ^2^Institute of Translational Pharmacology (IFT), National Research Council (CNR), Palermo, Italy

##### **Correspondence:** Maria De Filippo (maria.defilippo01@universitadipavia.it)

*Italian Journal of Pediatrics 2023*, **49(Suppl 1):**A15

**Background**

Anaphylaxis is a severe, potentially life-threatening allergic reaction. As for other allergic diseases, intrinsic heterogeneity has been reported in clinical presentation, severity, and triggers of anaphylaxis. Moreover, clinical predictors of food-induced anaphylaxis severity are still not available. Predicting who is the most at risk of severe anaphylaxis reactions would be of great interest to reducing anaphylaxis morbidity and mortality but remains challenging. This study primarily aims to describe the clinical heterogeneity of a cohort of children and adolescents with food-induced anaphylaxis using a novel nonsupervised statistical clustering analysis. The study also aims to identify potential clinical predictors of severity in this population.

**Materials and methods**

A monocentric retrospective study that included patients diagnosed with food-induced anaphylaxis according to the guidelines of the European Academy of Allergy and Clinical Immunology (EAACI), admitted to the Pediatric Allergy Clinic of the Policlinico San Matteo in Pavia from 2001 to 2021 were included. Demographic, clinical (severity of reaction, allergic comorbidities, and food triggers), diagnostic (in vivo, in vitro, and oral challenge tests), and therapeutic data were reviewed. Fifteen clinical and etiological variables were used as inputs for the clustering algorithm.

**Results**

137 patients with a mean age of 5,6 years. Food culprits were tree nuts (38%), milk (20%), egg (15%), peanut (15%), fish (8%), and other ailments (3%). Using Sampson’s severity grading system, most anaphylaxis reactions were of severity grade 4 (59%). Only 19% of patients in our study underwent intramuscular adrenaline therapy. Four phenotypes emerged from cluster analysis. Cluster 1 (39 patients) was characterized by mild to moderate reactions, history of atopy, milk allergy, and gastrointestinal manifestations; Cluster 2 (28 patients) was characterized by severe reactions, nonatopic comorbidities, and gastrointestinal and respiratory manifestations; Cluster 3 (51 patients) was characterized by severe reactions, moderate frequencies of allergic comorbidities, respiratory and gastrointestinal manifestation; Cluster 4 (19 patients) was characterized by severe reactions, history of atopy, peanut allergy and respiratory manifestations associated with gastrointestinal, neurological and cardiovascular manifestations.

**Conclusions**

Our cluster analysis confirms that children with a history of atopy and peanut allergy represent a distinct clinical phenotype, with a higher risk of developing severe anaphylaxis than children without other allergic comorbidities. Further and larger studies are needed to better define high-risk patients who can benefit from a personalized approach using innovative treatments such as oral allergen immunotherapy and biological drugs.

**Ethics approval**

The Ethical Committee approved this study (protocol number 0003241/22).

**References**

1. Muraro A, Worm M, Alviani C, Cardona V, DunnGalvin A, Garvey LH, et al. EAACI guidelines: Anaphylaxis (2021 update). Allergy. 2022;77:357-377.
2. Martelli A, Ippolito R, Votto M, De Filippo M, Brambilla I, Calvani M, et al. Acta Biomed. 2020 ;91:e2020005.

## A16. Psychological impact of Covid-19 pandemic on people with Primary Ciliary Dyskinesia

### Valeria Delle Cave^1^, Claudia Biondi, Maria Pia Riccio, Adele Corcione, Marilena Cipullo, Giuliana Ponte, Simona Basilicata, Melissa Borrelli, Francesca Santamaria

#### ^1^Department of Translational Medical Sciences, Federico II University, 80131 Naples, Italy

##### **Correspondence:** Valeria Delle Cave (val.dellecave@gmail.com)

*Italian Journal of Pediatrics 2023*, **49(Suppl 1):**A16

**Background**

Primary ciliary dyskinesia (PCD), a rare genetic disorder, is characterized by impaired mucociliary clearance and recurrent-to-chronic respiratory infections. Development of bronchiectasis with progressive loss of pulmonary function, fertility issues and situs viscerum inversus in 50% of cases (Kartagener syndrome) are the hallmarks of the condition. The primary aim of this study was to prospectively assess the PCD patients’ psychological burden and parental stress levels one year after the beginning of the pandemic period.

**Materials and methods**

At the Department of Translational Medical Science,University “Federico II”, Naples. Italy, a specialized center that provides care to children and adults with PCD was estabilished in 1993. We divided the PCD population in two age-based groups, <15 years-old (Group A) and ≥15 years-old (Group B). In Group A, PCD parents’ stress was assessed through the Italian version of the Parenting Stress Index-Short Form Questionnaire (PSI-SF) and in Group B with the Psychological General Well-Being Index Questionnaire (PGWBI). Data referring were obtained by email. The questionnaires were also administered to 27 age- and sex-matched controls.

**Results**

PCD patients included 10 subjects aged <15 years and 17 cases aged ≥15 years in the period May 2020-May 2021. Results showed that in 6/10 patients of Group A (60%),the total scores fall above the 50° percentile, indicating parental stress and were not significantly different than the same scores in controls (p>0.05). Comparing the results of the questionnaire administered in May 2020 with those of May 2021, were not significantly different (p>0.05). The results of the PGWBI questionnaire showed that 23% of Group B participants reached a total scores indicated moderate distress and in 17% severe distress. Compared with the control groups and with the results of the questionnaire administered in May 2020, no significant differences were valued (p>0.05). Nevertheless, comparing the scores obtained for each subscale, the percentage of subjects with clinically relevant scores associated to ‘depression’,‘general health,‘self control’and ‘vitality’was greater in the control group than in PCD.

**Conclusions**

Patients with PCD neither developed psychological distress one year after the beginning of the pandemic period. Likely,for patients with PCD,who had already taken preventive measures to limit the infections for their underlying condition, the restrictive measures imposed by the pandemic didn’t have an impact on their psichological well-being. On the other hand, in healthy subjects, they caused greater anxiety and depressive symptoms confirming the data in the literature. In conclusion, unlike the healthy population, subjects with chronic disease may be better able to use strategies to cope the stress conditions.

**Ethics Approval**

The study was performed in accordance with the Declaration of Helsinki for Human Research and approved by the Ethical Committee, Federico II University, Naples (protocol no. 275/20).

## A17. SARS-Cov2 infection in paediatric patients with cystic fibrosis

### Michela Deolmi, Cinzia Spaggiari, Valentina Fainardi, Giovanna Pisi, Susanna Esposito

#### Università di Parma, Dipartimento di Medicina e Chirurgia, Parma, Italy

##### **Correspondence:** Michela Deolmi (michela.deolmi@studenti.unipr.it)

*Italian Journal of Pediatrics 2023*, **49(Suppl 1):**A17

**Background**

Preliminary data from literature show that prevalence of Sars-Cov2 infection in patients with cystic fibrosis (CF) is similar compared to the general population, but the rate of hospitalization and the need for intensive care is higher [1,2]. This study aims to evaluate the course and outcomes of SARS-Cov2 infection in paediatric patients with cystic fibrosis.

**Materials and methods**

In this retrospective study, we collect medical history, clinical data and the results of lung function tests of paediatric patients with CF followed up at the Cystic Fibrosis Center of Parma. Included patients were diagnosed with SARS-Cov2 infection in the previous six months, between February 2020 and February 2022.

**Results**

We enrolled 15 CF patients (mean age 8.5 ± 3.9 years, range 1-14 years, 8 male) with previous SARS-Cov2 infection, diagnosed more frequently (n = 11, 73%) between November 2021 and January 2022. 67% of patients (n = 10) had at least one copy of the F508del mutation, 47% (n = 7) had pancreatic insufficiency and 27% (n = 4) had chronic respiratory colonization. In addition, two patients had CF-related diabetes and none had undergone lung transplantation. Most patients have been immunized for influenza (n = 12, 80%) and 47% (n = 7) for SARS-Cov2. (Table 1)

At the time of infection, 5 (33%) patients were asymptomatic, 8 (53%) had fever, 3 (20%) cough and 4 (27%) rhinitis. Other symptoms (pharyngitis, asthenia, myalgia, diarrhea, headache) were present in 4 (27%) patients. Nobody needed hospitalization or intensive care. The median duration of symptoms was 7.3 days (range 1-25).(Table 2)

Lung function indices remained substantially unchanged before and after SARS-Cov2 infection (mean FEV_1_ in the previous year: 78 ± 7%, mean FEV_1_ one month later: 75.5 ± 10.6%).

**Conclusions**

Contrary to expectations, paediatric cystic fibrosis patients in our cohort present a clinically mild SARS-Cov2 infection with no significant effect on lung function. More research, a larger sample and comparison with a control group are needed to better explain the long-term outcomes of SARS-Cov2 infection in paediatric patients with cystic fibrosis.

**Ethics Approval**

The study was approved by the Local Ethics Committee.

**References**

1. Naehrlich L, Orenti A, Dunlevy F, Kasmi I, Harutyunyan S, Pfleger A. et al. European Cystic Fibrosis COVID project group. Incidence of SARS-CoV-2 in people with cystic fibrosis in Europe between February and June 2020. J Cyst Fibros. 2021;20:566-577.
2. Jung A, Orenti A, Dunlevy F, Aleksejeva E, Bakkeheim E, Bobrovnichy V, et al. Factors for severe outcomes following SARS-CoV-2 infection in people with cystic fibrosis in Europe. ERJ Open Res. 2021;7:00411-2021.

**Table 1 (abstract A17):** **.** Population

Population (n. 15)	
Mean age (years±SD)	8.5 ± 3.9
Gender	8 M, 7 F
Pancreatic insufficiency, n (%)	7 (47)
Chronic respiratory colonization, n (%)	4 (27)
CF-related diabetes, n (%)	2 (13)
SARS-Cov2 immunization, n (%)	7 (47)

**Table 2 (abstract A17):** **.** Symptoms related to SARS-Cov2 infection in children with cystic fibrosis

Symptoms related to SARS-Cov2 infection in children with cystic fibrosis	
None, n (%)	5 (33)
Fever, n (%)	8 (53)
Cough, n (%)	3 (20)
Rhinitis, n (%)	4 (27)
Other (pharyngitis, asthenia, myalgia, diarrhea, headache), n (%)	4 (27)
Mean length of symptoms (days)	7,3

## A18. Long-term follow up of newborn screening for Pompe Disease

### Lilian Di Salvo, Vincenza Gragnaniello, Alberto B. Burlina

#### Division of Inherited Metabolic Diseases, Department of Diagnostic Services, University Hospital, Padua, Italy

##### **Correspondence:** Lilian Di Salvo (liliandisalvo@libero.it)

*Italian Journal of Pediatrics 2023*, **49(Suppl 1):**A18

**Background**

Pompe disease (PD) is a myopathy caused by a deficiency of the lysosomal acid alpha 1,4 glucosidase (GAA).

Early initiation of ERT in infantile-onset PD improves survival, reduces the need for ventilation and enhances patient quality of life. Newborn screening (NBS) is the optimal approach for early diagnosis and treatment of PD. Recently, newborn screening (NBS) for PD has been implemented around the world.

**Materials and methods**

From September 2015 to October 2021, 193,430 newborns performed dosing GAA by the MS/MS method.

If the mean of the enzyme activity values was confirmed to be low, a second DBS was requested. Newborns with abnormal enzyme results on the second round were referred to the Division of Inherited Metabolic Disease, Padua University Hospital, for confirmatory testing and clinical follow-up.

**Results**

Of the 193.430 newborns screened, 39 (F 17; M 22) had a low GAA enzyme activity in the DBS. Pompe disease was confirmed in 14 newborns. 3 were affected from infantile PD (IOPD, 1 positive cross-reactive immunologic material (CRIM), 2 CRIM negative), 11 children were affected by the late form (LOPD), 16 by pseudodeficiency and 4 carriers of a single GAA gene mutation, 2 children presented mutation of uncertain significance (VUS).

In our population total incidence of PD is 1: 13.816.

In IOPD patients, diagnosis occurred at 3-13 days of life and the ERT started at 5-15 days days. At the first visit, all IOPD patients had an increase in Glc4 and left ventricular mass (LVMI).

All IOPD patients presented with some developmental delay motor but have achieved autonomous walking and no one needs support ventilatory (age 2-4 years). The Glc4 progressively decreased. Patients with LOPD persist asymptomatic and follow annual monitoring. Nobody is in ERT.

**Conclusions**

Our case history confirms that detecting IOPD cases in the first weeks of life is essential for starting therapy and achieving optimal results. NBS is important for diagnosis and for starting therapy. Ethical questions persist for the neonatal diagnosis of LOPD forms.

**Consent to publish**

Written informed consent was obtained from a parent.

## A19. Chronic Recurrent Multifocal Osteomyelitis (CRMO): a case report

### Giulia Dodi, Maria Candelino, Luciana Breda, Francesco Chiarelli

#### Clinica Pediatrica, Chieti, Italy

##### **Correspondence:** Giulia Dodi (giulia.dodi16@gmail.com)

*Italian Journal of Pediatrics 2023*, **49(Suppl 1):**A19

**Background**

Joint pain is one of the most common symptoms that paediatricians commonly experience in children and adolescents [1]. Most of the time, the painful joint is a hip joint that often comes with protective lameness and reduced mobility [1]. This condition has broad differential diagnosis that includes very common and much rarer diseases [2,3].

**Case Report**

M., 10 years, was evaluated for left coxalgia. She had pain in left hip for two months, with nocturnal awakenings and limited movement, with no other symptom; pain was partially responsive to NSAIDs. She had a previous episode of coxitis of the right hip, with negative joint ultrasound and blood tests, at 7 years, treated with anti-inflammatory and antibiotic therapy with benefit. During physical examination she showed pain due to active and passive mobilization of the left hip, no signs of joint inflammation, painfulness on palpation of the sacral spinous processes. In view of clinical history and objectivity, we performed blood tests (ESR 34 mm/h; negative ANA, HLAB27, FR, anti-DNase and TAS), joint ultrasound of the hip with minimal left joint effusion and pelvic X-ray, which was negative. In consideration of red flags (e.g. chronic symptoms and nocturnal awakenings MRI of pelvis and spine were performed, too. MRI of the pelvis and lumbosacral spine showed multiple regions of altered signal of the spongy bone located at the level of the proximal diaphysis-metaphysis of the left femur, of the anteroinferior edges of D12 and anterior-superior of L5 and of the soma of S3 with nuanced impregnation after contrast medium. Peripheral venous smear and medullary aspirate excluded a possible onco-haematological cause, whereas bone scintigraphy, X-ray of the spine and clavicle excluded further localizations. M. was discharged with the diagnosis of Chronic Recurrent Multifocal Osteomyelitis (CRMO), with NSAIDs therapy. For persistence of symptoms, lumbosacral bone biopsy was organized in another institute; bone biopsy revealed non-specific inflammatory pattern and the diagnosis was confirmed. Within a couple of months, due to lack of control with NSAIDs, methotrexate was initiated. M. is still being monitored at our rheumatology centre.

**Conclusions**

CRMO is an autoinflammatory disease characterized by bone pain worsening at night, involving one or multiple sites, with or without fever and extra-skeletal symptoms [2,3]. It is an exclusion diagnosis: blood tests are not specific, MRI is crucial and often bone biopsy is necessary to rule out other diagnosis [2-4]. Therapy is not standardized: NSAIDs, glucocorticoids, DMARDs and biologics drugs are reported [2].

**Consent to publish**

Informed consent to publish has been obtained from the patients and her parents.

**References**

1. Yagdiran A, Zarghooni K, Semler JO, Eysel P. Hip pain in children. Dtsch Arztebl Int. 2020;117:72-82.
2. Menashe SJ, Aboughalia H, Zhao Y, Ngo AV, Otjen JP, Thapa MM, Iyer RS. The many faces of pediatric Chronic Recurrent Multifocal Osteomyelitis (CRMO): A practical location- and case-based approach to differentiate CRMO from its mimics. J Magn Reson Imaging. 2021;54:391-400.
3. Sato TS, Watal P, Ferguson PJ. Imaging mimics of chronic recurrent multifocal osteomyelitis: avoiding pitfalls in a diagnosis of exclusion. Pediatr Radiol. 2020;50:124-136.
4. Shah A, Rosenkranz M, Thapa M. Review of spinal involvement in Chronic Recurrent Multifocal Osteomyelitis (CRMO): What radiologists need to know about CRMO and its imitators. Clin Imaging. 2022;81:122-135.

## A20. Malnutrition in children with cerebral palsy: preliminary data from the VIGOUR Project

### Chiara Esposito^1^, Elena Scarpato^1^, Pietro Buono^2^, Antonella Anginoni^2^, Giovanna Affinito^2^, Tiziana Ciarambino^2^, Erasmo Miele^1^

#### ^1^Department of Translational Medical Sciences - section of Pediatrics AOU Federico II, Naples, Italy; ^2^General Direction for Health Protection and Coordination of the Regional Health System, Campania Region, Naples, Italy

##### **Correspondence:** Elena Scarpato (elenascarpato@hotmail.it)

*Italian Journal of Pediatrics 2023*, **49(Suppl 1):**A20

**Introduction**

VIGOUR is an international project funded by the European Community aiming at spreading good practices in integrated care. Campania Region and AOU Federico II of Naples have joined this project, focusing their activities on pediatric patients with infantile cerebral palsy (ICP) and gastrointestinal/nutritional disorders. The main goals include the creation of a hospital-territory network for the diagnosis and clinical management of children with ICP and nutritional/gastrointestinal disorders, and the creation of a registry of patients.

**Materials and methods**

For the creation of the registry, we started identifying patients with ICP and gastrointestinal/nutritional disorders followed-up at AOU Federico II of Naples, using the ICD9CM identification codes for ICP. We included patients aged between 2 and 18 years. Subjects were classified as having nutritional disorders in case of a weight Z-score < -1.65 and/or triceps skinfold thickness (SFT)<10°ct and/or enteral nutrition via a tube or percutaneous gastrostomy.

Subjects were classified as having gastrointestinal disorders in presence of gastroesophageal reflux disease (GERD) and/or chronic constipation.

**Results**

We identified 89 patients with ICP. Of these, 72 (80.1%) had nutritional/gastroenterological problems. Mean age of the included patients was 8.8±4.32 years (males 56.2%). Mean weight z-score was -4.41±1.56, mean stature z-score was -3.06±2.17, and mean BMI z-score was -3.66±2.79. Fifty percent of the subjects had poor weight gain, and 76.4% had a triceps SFT <10°ct. Concerning the feeding modalities, 69.4% were fed orally with natural foods, 8.3% were fed orally with natural foods and fortifiers, and 22.2% were on enteral nutrition. Of the 72 patients, 68% had dysphagia, 58.3% had chronic constipation, and 45.8% had GERD. Moreover, these three disorders were present simultaneously in 27.8% of cases, while 33.3% had two of the above disorders, and only 22.2% had only one disorder.

**Conclusions**

Our preliminary data confirmed that gastrointestinal and nutritional disorders are extremely common in patients with neurological impairment, yet in most subjects these issues are not part of the routine assessments and are treated in a promptly manner, resulting in a high prevalence of malnutrition. Therefore, healthcare providers involved in the care of subjects with ICP should be specifically trained to identify gastroenterological and nutritional disorders in this subgroup of patients.

**Ethics Approval**

The study involved only the creation of a clinical registry with data collected during standard clinical practice, so ethics committee approval is not required

## A21. Insulin T 60 in OGTT: a valuable tool for NAFLD screening in severely obese adolescents?

### Ludovica Fedi^1^, Alessandro Fierro^1^, Francesca Di Candia^1^, Irene Cuccurullo^2^, Sara Mobilia^2^, Federica Salerno^1^, Alberto Casertano^3^, Enza Mozzillo^1^, Adriana Franzese^1^

#### ^1^Regional Center of Pediatric Diabetes, Department of Translational Medical Sciences, Section of Pediatrics, Federico II University, Via Sergio Pansini 5, 80131 Naples, Italy; ^2^Regional Center of Pediatric Diabetes, Section of Pediatrics, Federico II University, Via Sergio Pansini 5, 80131 Naples, Italy; ^3^Pediatrics Unit, Rummo Hospital, Benevento, Italy

##### **Correspondence:** Alessandro Fierro (alessandro.fierro94@gmail.com)

*Italian Journal of Pediatrics 2023*, **49(Suppl 1):**A21

**Background**

Nonalcoholic Fatty Liver Disease (NAFLD) is a significant comorbidity in pediatric obesity. The increase of alanine transaminase (ALT) values and the Ultra Sonography (US) imaging are the currently noninvasive tools used for the diagnosis of NAFLD. The use of ALT detection is quite limited due to the lack of a universally accepted threshold caused by its variability related to age, sex, ethnicity, and lifestyle. Equally the US instrumental investigation has its own limits: operator-dependent, and difficulty in the evaluation of severe obesity. NAFLD is a marker of insulin-resistance and a predictor of complications such as type II diabetes and cardiovascular events. As reported in literature, insulin level time at 120 minutes from the oral glucose tolerance test (OGTT) is predictive of NAFLD in adults. The aim of the study is to identify if insulin levels in oral glucose tolerance test (OGTT) can be predictive of NAFLD in obese children.

**Materials and methods**

Children and adolescents of Caucasic ethnicity in puberal age affected by severe obesity (Age>11 years; BMI z-score >2) followed at our clinic from 2015 to 2020. Patients with chronic diseases, genetic syndromes and endocrinologic disorders were excluded. All patients received a standard clinical and laboratory evaluation and an OGTT test. NAFLD was diagnosed using US criteria and correlated to anthropometric variables and laboratory data. OGTT data were analyzed by ROC curve.

**Results**

119 severe obese children and adolescents (58 women, age 13,1 ± 1,75 years) were excluded. The OGTT of NAFLD patients showed significantly higher insulin levels at 60 minutes time (medium cut-off 122 μU/mL).

**Conclusions**

OGTT insulin level at 60 minutes time seams more predictive of NAFLD in obese children. Prospective studies with a larger sample of pediatric patients with obesity are needed to confirm these results.

**Ethical approval**

The present study was performed in accordance with the Helsinki declaration. The parents of each patient provided written informed consent. Ethical approval was not required due to the retrospective nature of the study.

## A22. The new face of cystic fibrosis: an example of genetic variants of uncertain significance

### Lucia Gerbaudo^1^, Elisa Romano^2^, Andrea Perna^1^, Andrea Savino^1^, Elisabetta Bignamini^3^

#### ^1^Università degli Studi di Torino, Torino, Italy; ^2^Segreteria Lega Italiana Fibrosi Cistica- AOU Città della Salute e della Scienza di Torino, Torino, Italy; ^3^AOU Città della Salute e della Scienza di Torino, Torino, Italy

##### **Correspondence:** Lucia Gerbaudo (lucia.gerbaudo@unito.it)

*Italian Journal of Pediatrics 2023*, **49(Suppl 1):**A22

**Background**

Cystic fibrosis (CF) is an autosomal recessive genetic disorder caused by CFTR’s (Cystic Fibrosis Transmembrane Regulator) gene mutation. At the moment, we know about 2200 gene mutations, which are classified into six different groups depending on their protein effect. L997F is a variant of the CFTR gene of uncertain significance. The carriers of this variant with another mutation causing disease, have different clinical pictures, from total absence of symptoms to CFTR related disease (eg recurrent pancreatitis, azoospermia, isolated bronchiectasis).

**Materials and methods**

We have studied 13 patients, aged between 6 and 10 years with the L997F variant in compound heterozygosis with another pathogenic variant, selected at the Pediatric Reference Center for diagnosis and treatment of Cystic Fibrosis of Piedmont-Valle d'Aosta.

The following data were analysed: cause of diagnosis, sweat test value, Body Mass Index (BMI) and FEV1% at 6 years, presence of bronchiectasis at imaging (RX or chest CT scan), pancreatic and hepatic function, respiratory infections and related use of antibiotics. The frequency of infections has been compared with two other populations of CF patients (13 + 13 patients), homogeneous in sex and age but genotypically different: one with CFTRrelated mutations and one with homozygous F508 mutation (CF with pancreatic insufficiency). The study was approved by the Intercompany Ethics Committee.

**Results**

Among the 13 selected patients, 9 were diagnosed thanks to neonatal screening pathway, 1 for genetic familiarity at 5 years old, 3 for respiratory symptoms with average age of 6.3.

The sweat test was negative in 3 patients (<30mEq/l of Cl) and positive in 2 (>60mEq/l of Cl), borderline in the remaining 8, with average mEq/l of Cl of 46.4. BMI averaged at 6-year was 16.0 (underweight). The FEV1% at 6 years averaged 99.2%. No subjects had bronchiectasis, pancreatic insufficiency, pancreatitis, liver disease or alteration of liver enzymes. Infections rate in this group of patients was lower than in CFTR-related mutations patients.

**Conclusions**

The presence of the L997F mutation, associated with a definitely pathogenetic variant, does not seem to induce symptoms of CF disease in school age children. It is however important that the Pediatrician regularly follows patients with uncertain clinical significant CFTR variants to find the possible delineation over the years of a CFTR related clinical pictures. Further studies are needed in the next years to rule out the pathogenicity of L997F, particularly in the population with altered or borderline sweat testing.

## A23. Diencephalic Syndrome: misleading clinical onset of glioma

### Carmela Gulizia^1^, Milena La Spina^2^, Giulio Pulvirenti^1^, Marta Arrabito^1^, Elisabetta Testa^1^, Marianna Strazzieri^1^, Miriana Ferrigno^1^, Daria La Cognata^1^, Federica Gullo^1^, Rachele Soma^3^, Andrea Di Cataldo^2^, Giovanna Russo^2^

#### ^1^Pediatrics Postgraduate Residence Program, Department of Clinical and Experimental Medicine, University of Catania, Catania, Italy; ^2^ Pediatric Hematology and Oncology Unit, Department of Clinical and Experimental Medicine, University-Hospital, “G. Rodolico-San Marco”, Catania, Italy; ^3^ Genetics Postgraduate Residence Program, Department of Clinical and Experimental Medicine, University of Catania, Catania, Italy

##### **Correspondence:** Milena La Spina (mlaspina@unict.it)

*Italian Journal of Pediatrics 2023*, **49(Suppl 1):**A23

**Background**

Diencephalic syndrome may be an atypical onset of low-grade cerebral glioma.

**Materials and methods**

Period 2017-2021: four pediatric cases of low-grade glioma and diencephalic syndrome.

**Case 1:** 3-year-old male, diagnosis of bulbar pilocytic astrocytoma. Symptoms: recurrent vomiting, weight <3 standard deviations (DS), normal linear growth. Latency between symptoms onset and diagnosis was 24 months.

**Case 2:** 9 month-old male, diagnosis of hypothalamic pilocytic astrocytoma. Onset symptoms: recurrent vomiting, weight <3 DS, normal linear growth; horizontal nystagmus appeared after 4 months from diagnosis. Latency between symptoms onset and diagnosis was 7 months.

**Case 3:** 15-year-old female, diagnosis of suprasellar pilocytic astrocytoma with extensive leptomeningeal dissemination in the brain and spinal cord. Progressive weight loss, << 3 DS weight, normal linear growth, selective feeding, hypogonadotropic hypogonadism. First diagnosis was anorexia nervosa; after two years inconstant headache and severe emaciation appear followed by vomiting, photophobia, dysarthria and paresthesia after a further year. Latency between symptoms onset and diagnosis was 4 years.

**Case 4:** 10-year-old female, diagnosis of diffuse suprasellar pilocytic astrocytoma. Mood and eating disorders, progressive weight loss, << 3DS weight, normal linear growth rate. Latency between symptoms onset and diagnosis was 1 year.

**Results**

Four cases of low-grade glioma and diencephalic syndrome are reported; three with diencephalic localization, one in an atypical bulbar site. Mean latency between symptom onset and diagnosis was 22.75 months. Currently, patient 1 and 2 have stable disease in second-line therapy, patient 3 has stable disease 22 months after the end of second-line therapy and patient 4 has stable disease in first-line therapy.

**Conclusions**

Diencephalic Syndrome is an uncommon cause of failure to thrive in early childhood characterized by severe emaciated body, poor weight growth with a normal caloric intake and linear growth, sometimes hyperactivity, vomiting, mood/food/ endocrinological disorders, in presence of hypothalamic/chiasmatic low grade glioma. The non-specificity of the onset symptoms common causes diagnostic delay, favoring the growth of the neoplasm and worsening of disease-free survival. In fact, three of the four cases described presented disease progression after the first line therapy. We want to draw attention of pediatricians to the rare hypothesis of a tumor-associated diencephalic syndrome in any child with undergrowth, adequate caloric intake, normal linear growth, whether or not associated with gastrointestinal disorders without evidence of gastrointestinal/endocrinological pathology so that they plan a careful clinical-neurological monitoring, carrying out a complete eye examination and possibly a brain magnetic resonance when the symptoms persist without diagnosis.

**Consent to publish**

Written informed consent for publication was obtained from the parents

## A24. Syndromic and non-syndromic atrioventricular canal defect: From anatomy to prognosis, retrospective study

### Michela Leotta^1^, Ivana Bringheli^1^, Michela Amatruda^1^, Francesca Aurora Meo^1^, Lilia Oreto^2^, Francesco De Luca^1^, Letteria Bruno^1^, Maria Pia Calabrò^1^

#### ^1^Department of Human Pathology of adults and of developmental age. UOSD of Pediatric Cardiology, University of Messina, Italia; ^2^Mediterranean Pediatric Cardiology Center-OPBG, Taormina, Messina, Italia

##### **Correspondence:** Michela Leotta (micheleotta1@gmail.com)

*Italian Journal of Pediatrics 2023*, **49(Suppl 1):**A24

**Background**

Atrioventricular canal defect (AVCD) is a congenital heart disease commonly occurring with Down's Syndrome and, to a lesser extent, with other genetic syndromes or as an isolated, non-syndromic cardiac defect. The aim of this study is to analyze the different anatomical conformations AVCD can acquire in syndromic and non-syndromic patients and to point out differences concerning surgical management and prognosis between the above-mentioned groups.

**Materials and methods**

The study is based on a retrospective examination of clinical data, electrocardiographic, echocardiographic, angiocardiographic evaluations and surgical reports collected in the database of the UOSD of Pediatric Cardiology of the University Hospital of Messina and of the Mediterranean Pediatric Cardiology Center-Bambino Gesù Pediatric Hospital of Taormina.

**Results**

70 patients were included in the study: 40 females (57%) and 30 males (43%). Of the patients, 32 (46%) were non-syndromic patients (group A); 33 (47%) had Down Syndrome (group B), and 5 (7%) with other syndromes (abbreviated as AS). Balanced form of complete AVCD was found in 8 out of 32 patients in group A (25%) and in 26 out of 33 in group B (79%) (p <0.001); the complete unbalanced AVCD in 8 out of 32 in group A (25%), and in 1 out of 33 in group B (3%) (p <0.01); the partial AVCD in 10 out of 32 in group A (31%), in 5 out of 33 in group B (15%) (p <0.1); and the intermediate AVCD in 6 out of 32 in group A (19%) and in 1 out of 33 of group B (3%) (p <0.05). In group A, 3-4 reoperations were needed, out of 6 non-syndromic patients (18.8%), 3 of who died. The latter represent the 60% of the overall cases of exitus (5/70 = 7.14%), along with a patient in group B and another one in group AS. Major arrhythmic complications concerned only group A. Mild-moderate failure of one and/or both reconstructed atrioventricular valves was the most frequent complication in all patients. The most demanding hemodynamic sequelae found was in KBG syndrome patient (AS). In this case, following the double patch anatomic correction, a severe stenosis of the reconstructed left atrioventricular valve occurred. A valvuloplasty was performed until replacement with mechanical prosthesis became necessary.

**Conclusions**

This study confirms the prevalence of complete balanced atrioventricular canal defect and its better prognosis in patients with trisomy 21, as shown in larger studies reported in the literature.

**Ethics approval**

The study did not need ethical approval because it is a retrospective observational study of clinical data, electrocardiographic, echocardiographic, angiocardiographic evaluations and surgical reports from human subjects collected in the database.

**Consent to publish**

It was not planned to acquire specific consent from the subjects included in the study because the research is a retrospective observational study and it was not possible to contact all patients to inform them. In addition, the abstract does not contain details related to individual participants, but related to a large number of patients.

## A25. Pneumothorax and Pneumomediastinum as complications of respiratory infections during the post covid-19 pandemic period

### Martina Mazzoni^1^, Martina Carucci^1^, Stefania Formicola^2^, Fabio Antonelli^2^, Barbara Borrelli^2^, Giovanna Gaudiello^2^, Paolo Cavaliere^2^, Anna Naclerio^2^, Eliana Brigante^2^, Mariachiara Petagna^2^, Pierluigi Vuilleumier^2^

#### ^1^Medicine, Surgery and Dentistry Department of “Scuola Medica Salernitana”, University of Salerno, Postgraduate School of Pediatrics, Baronissi, 84084, Italy; ^2^Complex Operative Unit of Pneumology and UTSIR of the Santobono-Pausilipon Children’s Hospital, Naples, 80122, Italy

##### **Correspondence:** Martina Mazzoni (mamazzoni@unisa.it)

*Italian Journal of Pediatrics 2023*, **49(Suppl 1):**A25

**Background**

A sudden stop of the usual epidemic peak of respiratory infections (RI) and a decrease in the spread of respiratory syncytial virus and respiratory viral pathogens was observed in March 2020 when the onset of severe acute respiratory syndrome coronavirus 2 (SARS-CoV-2) began. The aim of our study is to describe the frequency of RI after the beginning of the SARS-CoV-2 pandemic and to report the increase of pneumomediastinum (PNM) and pneumothorax (PNX) cases in the period from October 2021 to March 2022.

**Materials and Methods**

The discharge diagnoses of patients admitted to the UOC of Pneumology and Respiratory Sub-Intensive Care Unit of Santobono-Pausilipon Hospital, from October to March 2019, 2020 and 2021, were analysed, extrapolating cases of RI, PNM, PNX with and without comorbidity (asthma, chronic respiratory diseases). The data obtained were compared in relation to the year analysed and the timing of major or minor restrictions due to the pandemic of SARS-CoV-2.

**Results**

We identified three groups of study composed as followed: 64 patients in the period October 2019-March 2020 (pre-pandemic), 18 patients in the period October 2020-March 2021 (period of increased social restrictions) and 69 in the period October 2021-March 2022 (period of less social restrictions). In 2019-2020 period there were 62 cases of RI, 5 cases of PNM and/or PNX (60% associated with RI, 40% spontaneous); in 2020-2021 17 patients with RI and 1 with PNM (associated with comorbidity); in 2021-2022 64 cases of RI, 19 of PNM and/or PNX (68.4% associated with RI, 15.8% in patients with co-morbidity, 15.8% spontaneous). During the 2019-2020 period we reported 5 cases (20%) of PNM and/or PNX, during 2020-2021 1 case only (4%) and during 2021-2022 19 cases (76%). These data were consistent with increased trend in PNM and/or PNX cases in the period 2021-2022.

**Conclusions**

Our data confirm a drastic decrease in RI requiring hospitalization during the year 2020-2021 as already observed in the recent scientific literature. Recent scientific works have shown a lower circulation of new virus variants, due to social restrictions, and a viral competition between respiratory viruses and SARS-CoV-2. We also highlighted an increase of PNM and PNX cases during 2021-2022 if compared with 2020-2021 and 2019-2020. Most of these cases were associated with RI, suggesting a likely increase in virulence of virus or a higher susceptibility of paediatric patients who were apparently naive to respiratory viruses in the post-social restriction period.

**Ethical approval**

Ethics approval was not obtained due to the retrospective and anonymised approach to collect data, which did not change the clinical practice nor provoked any privacy leak for confidential data.

**Consent to publish**

Consent to publish the abstract was not obtained. This study does not contain any personal information that could lead to the identification of the patients.

## A26. Surgical antimicrobial perioperative prophylaxis for neonatal and pediatric population

### Laura Nicoletti^1^, Sara Monaco^1^, Sonia Bianchini^1^, Erika Rigotti^2^, Elena Carrara^3^, Francesca Opri^2^, Roberta Opri^2^, Caterina Caminiti^4^, Giorgio Conti^5^, Cinzia Auriti^6^, Daniele Donà^7^, Alessandro Inserra^8^, Laura Lancella^9^, Giorgio Piacentini^2^, Nicola Principi^10^, Simonetta Tesoro^11^, Elisabetta Venturini^11^, Fabio Mosca^12^, Alberto Villani^9^, Annamaria Staiano^13^, Susanna Esposito^1^

#### ^1^Pediatric Clinic, University Hospital, Department of Medicine and Surgery, University of Parma, Parma, Italy; ^2^Pediatric Unit, Department of Surgical Sciences, Dentistry, Gynecology and Pediatrics, University of Verona, Verona, Italy; ^3^Infectious Diseases Section, Department of Diagnostics and Public Health, University of Verona, Verona, Italy; ^4^Research and Innovation Unit, University Hospital of Parma, Parma, Italy; ^5^Pediatric ICU and Trauma Center, Fondazione Policlinico Universitario A. Gemelli IRCCS, Rome, Italy; ^6^ Neonatology and Neonatal Intensive Care Unit, IRCCS Bambino Gesù Children’s Hospital, Rome, Italy; ^7^Division of Paediatric Infectious Diseases, Department for Woman and Child Health, University of Padua, Padua, Italy; ^8^General Surgery Department, Bambino Gesu Children's Hospital, Istituto di Ricerca e Cura a Carattere Scientifico (IRCCS), Rome, Italy; ^9^ Paediatric and Infectious Disease Unit, Academic Department of Pediatrics, IRCCS Bambino Gesù Children’s Hospital, Rome, Italy; ^10^ Università degli Studi di Milano, Milan, Italy; ^11^Division of Anesthesia, Analgesia and Intensive Care, Department of Surgical and Biomedical Sciences, University of Perugia, Perugia, Italy; ^12^Neonatal Intensive Care Unit, Fondazione IRCCS Ca' Granda Ospedale Maggiore Policlinico, Università degli Studi di Milano, Milan, Italy; ^13^Department of Translational Medical Science, Section of Pediatrics, University of Naples “Federico II”, Naples, Italy

##### Correspondence: Laura Nicoletti

*Italian Journal of Pediatrics 2023*, **49(Suppl 1):**A26

**Background**

Surgical site infections (SSIs) represent a potential complication of any type of surgery, with a significant impact on mortality, morbidity and healthcare costs. Type of surgery, its duration, preoperative preparation and patient's conditions, influence the incidence and severity of SSIs. Perioperative administration of antibiotics, combined with other preventive measures, allows to reduce the occurrence of SSIs. Currently, data regarding the neonatal and pediatric populations are scarce. Therefore, SSIs prophylaxis is commonly based on single-center protocols.

**Materials and methods**

This consensus document was created using the RAND/UCLA (Research and Development Corporation of the University of California - Los Angeles) appropriateness method. A multidisciplinary group of experts composed of pediatricians, neonatologists, infectious diseases specialists, pediatric surgeons, anesthesiologists, pharmacologists, and microbiologists was selected from the main Italian scientific societies. A literature search was performed and a questionnaire on perioperative prophylaxis for neonatal and pediatric population was created using the Patient/Problem/Population–Intervention–Comparison/Control/Comparator–Outcome (PICO) model. The questionnaire, divided in different specific scenarios about a specific type of intervention and / or type of patient, was submitted on the online platform “REDCap” to a panel of experts, and they anonymously expressed opinions about the appropriateness of each individual intervention. Results were discussed collectively to reduce eventual disagreement, and later, all participants were asked to approve the recommendations in a second round during the following four weeks.

**Results**

The recommendations regarded the following surgical disciplines: cardiothoracic, abdominal, genitourinary, plastic, ophthalmology, orthopedic, ENT, maxillofacial and neurosurgery. Finally, two sections of the document were dedicated to general measures for SSIs prevention and to the management of perioperative prophylaxis of patients who have a high risk of SSIs due to specific starting conditions, pre-existing pathology or colonization by multi-resistant germs.

**Conclusions**

This consensus document, made with multidisciplinary contribution of experts belonging to the most important Italian scientific societies, represents a complete and up-to-date collection of recommendations regarding the perioperative prophylaxis for neonatal and pediatric patients. The application of shared protocols aims at optimizing the use of perioperative prophylaxis to reduce the incidence of SSIs and containing the antimicrobial resistance.

**Ethics approval**

Ethics approval is not required for a consensus document.

## A27. From obesity to type 2 diabetes mellitus: round trip

### Pamela Paglia^1^, Giuliana Valerio^2^, Maria Rosaria Licenziati^3^

#### ^1^Department of Medicine, Surgery and Dentistry "Scuola Medica Salernitana", University of Salerno, Salerno, Italy; ^2^Department of Movement Sciences and Wellbeing, University of Naples Parthenope, Naples, Italy; ^3^Obesity and Endocrine Disease Unit, Department of Neurosciences, Santobono-Pausilipon Children's Hospital, Naples, Italy

##### **Correspondence:** Pamela Paglia (pagliapamela90@gmail.com)

*Italian Journal of Pediatrics 2023*, **49(Suppl 1):**A27

**Background**

Children and adolescents with obesity are exposed to physical and psychosocial complications which tend to worsen in adulthood. Type 2 diabetes mellitus (T2DM) is one of the most feared endocrine-metabolic complications [1,2,3]. Here is described the case of a patient suffering from severe obesity and T2DM regressed only after significant reduction in excess weight.

**Case report**

E.R., 10 years old, shows complicated severe obesity and family history of diabetes mellitus in first and second degree relatives. Obstructive sleep apnea reported. At the clinical-auxological evaluation: weight 122 kg, height 151 cm, BMI 53 kg/m2 (SDS +2.89), waist circumference 120 cm, waist circumference/height ratio 0.79, blood pressure 130/90 mmHg, acanthosis nigricans on neck and abdomen, striae rubrae, adipomastia and limitation to walking. Finding of hypertransaminasemia supported by ultrasound evidence of severe hepatic steatosis. The assessment of basal glycaemia and glycaemia after oral glucose load (OGTT) highlights a picture of prediabetes: fasting glycaemia 108 mg/dl, glycaemia after 2 hours 173 mg/dl, glycated hemoglobin (Hb1Ac) 6.1%. After 18 months worsening of auxological parameters (BMI 56 kg / m2; SDS +3.08) and laboratory tests: fasting glycaemia 184 mg/dl, fasting insulinemia 9 μU/ml, Homeostasis Model Assessment of Insulin Resistance (HOMA-IR) 4, Hb1Ac 8%, glycosuria 300 mg/dl, glycaemia and insulinemia after OGTT clearly pathological (Table 1).

The biochemical parameters of autoimmunity and basal C-peptide are normal; genetic screening for monogenic diabetes is negative. T2DM secondary to severe obesity is diagnosed and a multidisciplinary therapeutic approach is started: educational, dietary-nutritional, rehabilitative (Alter-G antigravity treadmill) and pharmacological (Metformin for 12 months). After 6 months from the suspension of oral hypoglycemic therapy, the boy shows a notable reduction in excess weight (BMI 39 kg/m2; SDS +2.66), blood pressure and hepatic steatosis, normal blood glucose values, improvement in obstructive sleep apnea, dysfunctional behaviors and quality of life.

**Conclusions**

The early diagnosis of physical and psychological complications of pediatric obesity is important, for the reversibility of most of them through multidisciplinary interventions aimed at reducing adipose mass. The treatment of obesity, especially if severe and/or complicated, must continue over the long term, with periodic evaluations of the results obtained and any changes to the personalized treatment plan [1,4,5].

**Consent to publish**

Written informed consent was obtained from the patient's parents for publication of this case report.

**References**

1. Valerio G, Licenziati MR, Manco M, et al. Conseguenze dell'obesità sulla salute del bambino e dell'adolescente. Minerva Pediatr. 2014;66:381-414.
2. Zeitler P, Fu J, Tandon N, et al. Type 2 diabetes in the child and adolescent. Pediatr Diabetes. 2014;15 Suppl 20:26-46.
3. Lorini R, D’annunzio G, Minuto N, et al. Il diabete mellito non autoimmune in età pediatrica. G It Diabetol Metab. 2010;30:172-183.
4. Grugni G, Licenziati MR, Valerio G, et al. The rehabilitation of children and adolescents with severe or medically complicated obesity: an ISPED expert opinion document. Eat Weight Disord. 2017;22:3-12.
5. Seburg EM, Olson-Bullis BA, Bredeson DM, et al. A Review of Primary Care-Based Childhood Obesity Prevention and Treatment Interventions. Curr Obes Rep. 2015;4:157-73.

**Table 1 (abstract A27):** **.** Glycaemia and insulinemia after oral glucose load (OGTT)

OGTT	Glycaemia	Insulinemia
Time 0’	87	25.1
Time 30’	137	56.5
Time 60’	177	50.6
Time 90’	199	95.9
Time 120’	220	125.1

## A28. Cholelithiasis in infants: the relevance of medical history

### Paola Pagliara^1^, Viola Santi^1^, Francesca Giffoni^1^, Francesca Olivero^1^, Francesca Rivano^1^, Angelo Tropeano^2^, Costantino De Giacomo^3^, Chiara Moretti^3^

#### ^1^Department of Pediatrics, Foundation Policlinico San Matteo, University of Pavia, Pavia, Italy; ^2^Department of human pathology of adult and developmental age “G.Barresi”, University of Messina, Messina, Italy; ^3^Department of Pediatrics, ASST Grande Ospedale Metropolitano Niguarda, Milan, Italy

##### **Correspondence:** Paola Pagliara (paola_pagliara@libero.it)

*Italian Journal of Pediatrics 2023*, **49(Suppl 1):**A28

**Background**

A. K. was born full term after a normal pregnancy, who presented jaundice at 24 hours of life. That resolved after phototherapy, subsequently the level of maternal anti-B antibodies was found to be positive. At 2 months of life A. K. was admitted to hospital for the appearance of acholic stools despite of a state of complete well-being.

**Materials and Methods**

At the beginning of the hospitalisation A. K. was not jaundiced and his urine were normochromic, with bilirubin levels in range for age. An abdominal ultrasound scan was performed on the second day of hospitalization to exclude anatomical anomalies in the biliary tract. Blood tests included G6PD status and a peripheral blood smear to rule out possible haemolytic causes. Serology and antigen of hepatitis viruses, alpha-1-antitrypsin levels and a detailed perinatal medical history were performed to exclude the major causes of cholestasis of the paediatric age.

**Results**

The abdominal ultrasound scan revealed gallbladder distension with thickened walls, initial dilation of left intrahepatic biliary system, dilation of the common bile duct and echogenic material indicative of intrahepatic cholelithiasis. Therapy with both ursodeoxycholic acid and vitamin K was started due to the possible haemolysis according to the medical history of A. K., the absence of clinical signs and symptoms before the appearance of pale stools and, most of all, the results of ultrasound scan. In the next days blood tests and ultrasound scans were performed frequently until the reappearance of normal stools after two days of medical therapy. Blood tests ruled out the most common causes of chronic haemolysis and infections; also, levels of alpha-1-antitrypsin were in range. Anisocytosis and stomatocytosis of red blood cells were discovered at peripheral blood smear, therefore, a haematological follow up was started.

**Conclusions**

In the differential diagnosis of cholelithiasis in infants it is essential to rule out biliary atresia in order to proceed with surgical intervention (Kasai operation) before 2 months of age [1,2]. In our case, the medical history of haemolysis, the period without signs and symptoms between birth and the onset of cholestasis in association to the ultrasound findings of a dilated common bile duct and cholelitiasis have been of primary importance for the diagnosis. This case report shows that cholelithiasis in infants has several causes in which the major predisposing factors are prematurity, infections or neonatal haemolysis (AB0 incompatibility) [3,4].

**Consent to publish**

Written informed consent has been collected from the patient’s parents for publication.

**References**

1. Maggiore G, Caprai S. La diagnosi di ittero colestatico nel neonato. Medico e Bambino 999;18(3):157-161.
2. Sokol RJ, Shepherd RW, Superina R, Bezerra JA, Robuck P, Hoofnagle JH. Screening and outcomes in biliary atresia: summary of a National Institutes of Health workshop. Hepatology. 2007 ;46:566-81.
3. Catassi C, “La litiasi biliare” in Manuale SIGENP di gastroenterologia ed epatologia pediatrica, 2^nd^ edition, 2020, Il Pensiero Scientifico Editore, pag 389-392.
4. Rossi G, Cirillo F, Sciveres M, Riva S, Ricotta C, Spada M, et al. Calcolosi biliare: non solo per adulti. Medico e Bambino. 2015;34:495-503.

## A29. Dysbiosis in children with neurological impairment and long-term enteral nutrition

### Martina Chiara Pascuzzi^1^, Valeria Calcaterra^1^ , Simona Panelli^2^ , Francesco Comandatore^2^ , Elisa Borghi^3^, Claudio Bandi^4^ , Gloria Pelizzo^1,5^ , Gian Vincenzo Zuccotti^1,2,5^ , Elvira Verduci^1,3^

#### ^1^Department of Pediatrics, “Vittore Buzzi” Children’s Hospital, Milan, Italy; ^2^Pediatric Clinical Research Center "Invernizzi", Department of Biomedical and Clinical Sciences "L.Sacco", University of Milan, Milan, Italy; ^3^Department of Health Sciences, University of Milan, Milan, Italy; ^4^Department of Biosciences and Pediatric Clinical Research Center "Romeo Ed Enrica Invernizzi", University of Milan, Milan, Italy; ^5^Department of Biomedical and Clinical Science, “L. Sacco”, University of Milan, Milan, Italy

##### **Correspondence:** Martina Chiara Pascuzzi (martina.pascuzzi@unimi.it)

*Italian Journal of Pediatrics 2023*, **49(Suppl 1):**A29

**Background**

Long-term enteral nutrition (LTEN) can lead to alterations in the gut microbiota (GM). The aim of this is to assess the presence of dysbiosis in the paediatric population with severe neurological impairment (NI) and LTEN.

**Materials and methods**

The 29 children (15M-14F, 2-18 years) enrolled had a NI (GMFCS V) and were fed through gastrostomy with a semi-elemental formula (1Kcal/ml) without fibre. The control group consisted of 13 healthy children (2-14 years). Weight, height, body segment length (Stevenson's method), waist circumference, body mass index (BMI) and pubertal stage (according to Marshall and Tanner) were evaluated. Blood samples for metabolic indices (insulin, total and HDL cholesterol, triglycerides, transaminases and gamma-glutamyl transpeptidase) were collected in the morning after an overnight fast. Insulin resistance was assessed using HOMA-IR and altered insulin sensitivity was defined as a HOMA-IR value>97.5th percentile for age, gender and pubertal stage. By analysing DNA extracted from faecal samples, the taxonomic composition of the GM was assessed.

**Results**

Comparisons of GM composition revealed a clear differential clustering of bacterial taxa between children with NI under LTEN and age/sex-matched healthy subjects (p-value<0.05 Wilcoxon test).

Children with NI presented the following increased taxa: the classes *Fusobacteriia* (2x10^-9^) and *Betaproteobacteria* (6.8x10^-8^); the orders *Fusobacteriales* (2x10^-9^), *Burkholderiales* (class:Betaproteobacteria, 6.8x10^-8^), *Enterobacteriales* (class: *Gammaproteobacteria*, 5.8x10^-4^) and *Synergistales* (class: *Synergistia*, 2x10^-5^); the genera *Parabacteroides* (1.5x10^-7^), *Fusobacterium* (3.4x10^-9^), *Klebsiella* (2x10^-7^, belonging to *Enterobacteriales*), *Cloacibacillus* (4.1x10^-9^ belonging to *Synergistales*) and *Sutterella* (3.7x10^-4^, belonging to *Burkholderiales*).

However, the significantly depleted taxa were the phylum *Firmicutes* (8.3x10^-6^), and within it, the class *Clostridia* (7.5x10^-9^) and the order *Clostridiales* (8.9x10^-9^). Within *Clostridiales*, the families *Lachnospiraceae* (1.7x10^-3^) and *Ruminococcaceae* (5.6x10^-5^). Within *Ruminococcaceae*, the genera *Faecalibacterium* (4.6x10^-7^) and its close relative, and short chain fatty acid (SCFA) producer *Gemmiger*. Other taxa under-represented in NI children comprehend *Bifidobacterium* (phylum: *Actinobacteria*, 1.2x10^-4^) and its higher taxonomic rankings.

Finally, based on insulin resistance and insulin sensitivity, there were no significant differences in the bacterial composition of the GM of children with NI.

**Conclusions**

Analysis of the bacterial composition of the GM reveals a deep dysbiosis. "Differential clustering" depends on NI and LTEN. The Protective bacterial taxa appear severely reduced (Faecalibacterium, Gemmiger), while taxa including known (Gammaproteobacteria and Klebsiella) or emerging (Synergistales, Cloacibacillus and Fusobacterium) pathogens are considerably increased. Lastly, Facealibacterium has been proposed as a diagnostic and prognostic biomarker of inflammatory bowel disease. These results lay a solid foundation for interventional clinical studies which could modulate the GM of children with NI by administering biotics.

**Acknowledgements**

Thanks to Fondazione Romeo and Enrica Invernizzi (Milan, Italy) for their extraordinary support.

**Ethics Approval**

The study was approved by ARNAS Civico-Di Cristina-Benfratelli, approval number 354 Civico 2016.

**Consent to publish**

Written informed consent to participate in this study was provided by the participants' legal guardian/next of kin.

## A30. Elaxacaftor/tezacaftor/ivacaftor in patients aged 12 – 18 years with cystic fibrosis: A single center experience

### Angela Pepe^1,2^, Carmela Colangelo^3^, Sergio Manieri^3^, Claudia Mandato^4^, Donatello Salvatore^2^

#### ^1^Department of Medicine, Surgery and Dentistry, "Scuola Medica Salernitana”, Postgraduate School of Pediatrics, University of Salerno, Baronissi, Italy; ^2^Cystic Fibrosis Center, San Carlo Hospital, Potenza, Italy; ^3^Department of Pediatrics, San Carlo Hospital, Potenza, Italy; ^4^Department of Medicine, Surgery and Dentistry "Scuola Medica Salernitana”, University of Salerno, Baronissi (Salerno), Italy

##### **Correspondence:** Angela Pepe (angpepe01@gmail.com)

*Italian Journal of Pediatrics 2023*, **49(Suppl 1):**A30

**Background**

In the last decade, the availability of Cystic Fibrosis Transmembrane conductance Regulator (CFTR) modulators has changed the clinical history of Cystic Fibrosis (CF).

Elexacaftor/Tezacaftor/Ivacaftor (E/T/I) is the most recently approved CFTR modulator for the treatment of CF in patients ≥ 12 years of age with at least one F508del mutation. It consists of two correctors (Elexacaftor and Tezacaftor) and one potentiator (Ivacaftor). We describe the effectiveness and safety of the treatment with E/T/I in patients aged 12 – 18 years at the CF Center of the Region Basilicata (Southern Italy).

**Materials and Methods**

Data from patients aged 12 – 18 treated with E/T/I were reviewed. The outcomes included: lung function expressed by forced expiratory volume in the first second (FEV1), sweat chloride concentration (SCC), nutrition expressed by Body Mass Index (BMI) z-score, number of pulmonary exacerbations (PEx), cystic fibrosis questionnaire-revised (CFQ-R) respiratory domain score, and safety. Data at baseline and after 1, 3-6 months of treatment were analyzed.

**Results**

10 patients [5M; median age 167 months (range 78-208 months)], were included in this study. The mean (SD) percent predicted (pp) FEV_1_ was 70,66% (20,76) at baseline and increased to 93,64% (16,70) after one month of treatment. The mean absolute ppFEV_1_ improvement was 22,98% (CI 95%: 17,61-28,35) after 1 month of treatment with E/T/I and 24,73% after 3-6 months (CI 95%: 15,37-34,09) (Figure 1).

The overall mean (SD) SCC was 95,6 (10,71) mmol/L at baseline, and it decreased to 62,2 (15,25) mmol/L after 1 month of E/T/I. The mean absolute change from baseline was −40,1 mmol/L (95% CI −51,75 to −28,45) after 1 month. The mean (SD) BMI z-score at baseline was −0,65 (0,89); it progressively increased to −0.28 (0.81) after 1 month and 0,22 (0,69) after 3-6 months. The mean absolute change was 0,37 (CI 95%: 0,25 -0,49) after 1 month and 0,82 (CI 95%: 0,54-1,10) after 3-6 months. The CFQ-R domain score quickly improved from a median baseline value of 63,89 to 94,44 after 1 month. No patient had PEx during follow-up period. An overall good safety profile was observed with no adverse drug reaction.

**Conclusions**

Treatment with E/T/I was effective and safe in pediatric patients with CF, determining an improvement in lung function and nutrition and the reduction of both PEx and burden of illness.

**Ethical approval**

This observational, retrospective study was notified to the local Ethics Committee on 2022, March 15. The research was conducted according to the Declaration of Helsinki regarding the Ethical Principles for Medical Research Involving Human Subjects.

**Consent to publish**

The informed consent to allow the use of anonymous clinical data for research purposes was obtained for all patients (or from their legal guardian).

**Fig. 1 (abstract A30):**
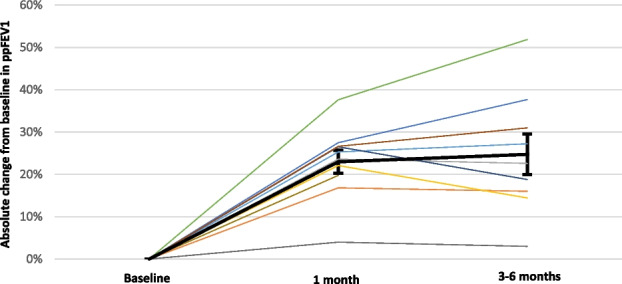
**.** Absolute change from baseline in ppFEV1 during follow-up. Colored lines show individual data. Mean values are shown by black bold line; the error bars indicate the standard error of the mean

## A31. Minipuberty in born small for gestational age infants: a case control prospective pilot study

### Giorgia Pepe^1,2^, Mariarosa Calafiore^3^, Maria Rosa Velletri^3^, Domenico Corica^1^, Tommaso Aversa^1^, Angela Alibrandi^4^, Malgorzata Wasniewska^1^

#### ^1^Department of Human Pathology of Adulthood and Childhood, University of Messina, Messina, Italy; ^2^Department of Biomedical, Dental, Morphological and Functional Imaging Sciences, University of Messina, Messina, Italy; ^3^Neonatology Unit, Bianchi-Melacrinò-Morelli Hospital, Reggio Calabria, Italy; ^4^Department of Economics, University of Messina, Messina, Italy

##### **Correspondence:** Giorgia Pepe (giorgiapepe23@gmail.com)

*Italian Journal of Pediatrics 2023*, **49(Suppl 1):**A31

**Background**

Minipuberty (MP) is still not well defined in small for gestational age (SGA) infants, due to controversial literature data. The present case-control, prospective study aims to evaluate MP in SGA infants, both preterm and full-term, compared with those born adequate for gestational age (AGA), during the first year of life.

**Materials and methods**

The study population included 33 SGA newborns (group A), 21 of which full-term (subgroup A1) and 12 preterm (subgroup A2). The control group (B) consisted of 27 AGA newborns, 17 of which full-term (subgroup B1) and 10 preterm (subgroup B2). All the participants were born in the same hospital and period. Sick newborns were excluded from the study. Periodic follow-up included growth parameters, FSH, LH, and Estradiol (E2) or Testosterone (T) serum levels at 3, 6 and 12 months.

Written informed consent was obtained earlier from the children’s parents.

**Results**

In the overall study population, the gonadotropin surge reached greater increase of LH in males at 3 months of age (p<0.001) and FSH in females at 3, 6 and 12 months (p<0.001), irrespectively of gestational age (GA). LH/FSH ratio remained higher in boys during the entire follow-up (p<0.001). Among the males: 1)T at 3 months was higher in subgroup A2 vs A1(p=0.001), and correlated negatively with GA, length and weight at birth (p<0.05); 2)LH was higher in subgroup B2 vs B1 at 6 months (p=0.003), and in group A vs B at 12 months (p=0.03); 3)LH/FSH ratio was increased in subgroup B2 vs B1 (p=0.004) at 6 months. Among the females: 1)E2 levels at 6 months were higher in subgroup B2 vs B1 (p<0.05), and negatively correlated with GA (p=0.015) and weight gain (p<0.05); 2)LH at 6 months and LH/FSH ratio at 3 months were increased in subgroup A2 vs A1 (p=0.03). Overall preterm boys, either SGA or AGA, displayed higher levels of T at 3 months (p=0.001) and LH at 3,6 and 12 months (p<0.05), together with increased LH/FSH ratio at 6 months (p=0.001). Preterm girls, either SGA or AGA, exhibited higher LH/FSH ratio at 3 and 6 months than full-term girls (p<0.05).

**Conclusions**

Irrespectively of GA, MP occurred with a typical sexual dimorphism and exhibited sex-specific correlations between hormones and perinatal parameters. The condition of SGA and prematurity seemed to enhance and protract MP over time in both sexes, suggesting that prenatal growth failure might influence hypothalamic-pituitary-gonadal axis activation.

## A32. Risk of SARS-CoV2 transmission and epidemiological characteristics of newborns born to women with COVID-19 infection: A retrospective observational study

### Mirella Petrisano, Giulia Ranucci, Giovanni Russo, Maria Scavone, Aurora Dinatale, Simona Pesce

#### Neonatal Intensive Care Unit, San Carlo Hospital, Potenza, Italy

##### **Correspondence:** Mirella Petrisano (mirella.petrisano@gmail.com)

*Italian Journal of Pediatrics 2023*, **49(Suppl 1):**A32

**Background**

The COVID-19 pandemic, emerged from China in December 2019, caused more than 250.000 deaths. Nevertheless, there are few studies in literature investigating the epidemiological characteristics and the risk of transmission in newborns exposed to SARS-CoV-2.

**Materials and methods**

We conducted a retrospective observational study enrolling pregnant women diagnosed with COVID-19 at the moment of the delivery at AOR San Carlo (Potenza, Italy) between February 22, 2020, and February 22, 2022. Parents provided informed consent. The newborn management was according to COVID-19 guidelines of the national neonatal society. Neonates were tested for SARS-CoV-2 by use of real-time PCR (RT-PCR) on nasopharyngeal swabs taken at 0 and 48 hours. The aim of this study was to evaluate the risk of perinatal transmission of COVID-19 and the clinical and epidemiological characteristics of the newborns.

**Results**

We enrolled 74 pregnant women with COVID-19 and 74 newborns. The rate of vaginal delivery was 60.8%. The mean gestational age at delivery was 38 weeks and 6 days (SD ± 1.98).

The percentage of term births was considerably higher (74.3%) compared to preterm births (1.4% <30 weeks, 2.7% between 30-34 weeks, 21.6% between 35-37 weeks gestation). The 5.4% of the newborns were admitted to NICU. Moreover, 83.8% of the newborns weighted appropriate for gestational age, 12.2% were small for gestational age and 4% were large for gestational age. The 6.8% (5/74) of the newborns tested positive for nasopharyngeal RT-PCR; 2 neonates tested positive to the first nasopharyngeal swab and negative to the second test, 3 neonates tested positive to the second nasopharyngeal swab. None of the neonates had symptoms of COVID-19.

**Conclusions**

In our study the trend in caesarean section was lower than worldwide data (70-85%) [1]; nowadays there are no universal recommendations for preferred delivery mode in women infected with COVID-19. Sars-CoV-2 positivity rate on the first swab in newborns was not found to be according to previous studies that did not confirm the transplacental transmission of the infection [2]; however, the subsequent negative swab suggest that false positivity or postnatal transmission were more likely. In conformity with literature , affected neonates were asymptomatic, confirming the benign course of the infection [3].

**References**

1. Bellos I, Pandita A, Panza R. Maternal and perinatal outcomes in pregnant women infected by SARS-CoV-2: A meta-analysis. Eur J Obstet Gynecol Reprod Biol. 2021;256:194-204.
2. Chi J, Gong W, Gao Q. Clinical characteristics and outcomes of pregnant women with COVID-19 and the risk of vertical transmission: a systematic review. Arch Gynecol Obstet. 2021;303:337-345.
3. Salvatore CM, Han JY, Acker KP, Tiwari P, Jin J, Brandler M, Cangemi C, Gordon L, Parow A, DiPace J, DeLaMora P. Neonatal management and outcomes during the COVID-19 pandemic: an observation cohort study. Lancet Child Adolesc Health. 2020;4:721-727.

## A33. A case of recurrent acute pancreatitis on a genetic basis

### Francesca Rivano^1^, Francesca Giffoni^1^, Francesca Olivero^1^, Paola Pagliara^1^, Viola Santi^1^, Angelo Tropeano^2^, Elena Altieri^3^, Laura Cazzaniga^4^, Ugo Antonio Aristide Cavallari^4^, Daniela Graziani^4^, Costantino De Giacomo^3^

#### ^1^Paediatric Clinic, Fondazione IRCCS Policlinico S. Matteo, University of Pavia, Italy; ^2^Department of Human Pathology of Adult and Developmental Age G. Barresi, University of Messina, Italy; ^3^Maternal and Child Department, S.C. Paediatrics, ASST Grande Ospedale Metropolitano Niguarda, Milano, Italy; ^4^Department of Medical Genetics, ASST Grande Ospedale Metropolitano Niguarda, Milano, Italy

##### **Correspondence:**Francesca Rivano (francesca.rivano@libero.it); Francesca Giffoni (francesca.giffoni@outlook.it)

*Italian Journal of Pediatrics 2023*, **49(Suppl 1):**A33

**Background**

Y. is a 6 year old boy born in Egypt to non-consanguineous parents. His past medical history showed nothing relevant. No family history of pancreatitis was recorded. He was admitted to the Paediatrics Unit of Niguarda Hospital in December 2021 for a first episode of acute pancreatitis (amylase and lipase >10 times normal; abdominal ultrasound picture of minimal inhomogeneity of the head of the pancreas with a surrounding fluid stratum); microbiological and immunological investigations were normal. He was discharged after intravenous hydration, pain relief therapy and fasting, with complete resolution of his symptoms. In the following weeks, he presented two further episodes of acute pancreatitis which led to re-hospitalization.

**Materials and Methods**

An abdominal MRI was performed in order to define pancreatic morphological features, which showed a picture of pancreas divisum without dilatation of the main pancreatic duct. To quantify the pancreatic functional reserve, faecal elastase was assayed, which was found to be normal. After the resolution of the acute picture, endoscopic retrograde cholangio-pancreatography was performed (ERCP) and pancreatic sphincterotomy, followed by leakage of multiple protein plugs. In consideration of recurrent episodes of pancreatitis, a blood sample was collected to search for predisposing genetic alterations.

**Results**

NGS sequencing of genes related to recurrent pancreatitis showed the presence of two variants in compound heterozygosity in the CFTR gene (S158T; R334W). Sweat test was performed for this finding, which was negative.

**Conclusions**

Acute recurrent pancreatitis (ARP) and chronic pancreatitis (CP) in childhood are rare entities and still poorly known [1]. Predisposing factors are largely unknown, but all studies recognize the central role of genetics. In particular, PRSS1 and CFTR mutations are the most commonly associated with CP, followed by SPINK1, CTRC, CASR, CPA1 [2]. There are also obstructive, traumatic, infectious and metabolic causes, or concomitant causes [3]. In the classification of PRAs it is always recommended to perform a sweat test, even in the presence of negative neonatal screening, in order to exclude mild forms of cystic fibrosis (CF) [4]. In the literature, R334W is known as causative of mild forms of CF (class IV-V). Paradoxically, genotypes associated with mild forms of CF have a higher risk of causing recurrent pancreatitis [6]. The S158T variant is rare, considered potentially pathogenic as it has been observed in patients with a positive screening test positive [5]. In the present case, the presence of a morphological alteration (pancreas divisum) and the compound heterozygosity for the two mutations, appear to contribute to the development of ARP.

**Consent to publish**

Written informed consent to publish has been obtained from the parents of this patient.

**References**

1. Gariepy CE, Heyman MB, Lowe ME, Pohl JF, Werlin SLet al. Causal evaluation of acute recurrent and chronic pancreatitis in children: Consensus from the INSPPIRE Group. J Pediatr Gastroenterol Nutr. 2017;64:95-103.
2. Whitcomb DC. “Genetic risk factors for pancreatic disorders. Gastroenterology. 2013;144:1292-302.
3. Lucidi V, Alghisi F, Dall'Oglio L, D'Apice MR, Monti L, De Angelis P, et al. The etiology of acute recurrent pancreatitis in children: a challenge for pediatricians. Pancreas. 2011;40:517-21.
4. Bai HX, Lowe ME, Husain SZ. What have we learned about acute pancreatitis in children? J Pediatr Gastroenterol Nutr. 2011;52:262-70.
5. McGinniss MJ, Chen C, Redman JB, Buller A, Quan F, Peng M, et al. Extensive sequencing of the CFTR gene: lessons learned from the first 157 patient samples. Hum Genet. 2005;118:331-8.
6. Chee Y. Ooi, Peter R. Durie. Cystic Fibrosis Transmembrane Conductance Regulator (CFTR) gene mutations in pancreatitis. J Cyst Fibros. 2012;11:355-362.

## A34. Nutritional outcome at 1 year after percutaneous gastrostomy placement, in pediatric patients with neurological impairment

### Simona Salomone, Elena Scarpato, Massimo Martinelli, Mariarosaria Serra, Erasmo Miele

#### Department of Translational Medical Science, Section of Pediatrics, University of Naples “Federico II”, Italy

##### **Correspondence:** Erasmo Miele (erasmo.miele@unina.it)

*Italian Journal of Pediatrics 2023*, **49(Suppl 1):**A34

**Background**

Children with neurological impairment frequently show malnutrition, secondary to dysphagia and gastrointestinal disorders [1]. Indications for the placement of an endoscopic percutaneous gastrostomy (PEG) include episodes of aspiration, malnutrition and intolerance to nutrition via nasogastric tube [2]. The aim of our study was to define the nutritional outcome after 1 year from the placement of a PEG, in pediatric patients with neurological impairment.

**Materials and methods**

In this retrospective study, data from neurologically impaired subjects aged 2 to 18 years who underwent a PEG placement between January 2016 and December 2020 at the Department of Pediatrics of the AOU Federico II of Naples, were collected. For each patient, the following data were recorded at baseline and 1 year (T1) after the placement of the PEG: demographic data, auxological parameters, mid upper arm circumference (MUAC), waist circumference, and triceps skinfold thickness (SFT).

**Results**

Seventeen children (35% males) were enrolled, of whom 10 (59%) had infantile cerebral palsy and 7 (41%) had a neurological impairment related to other conditions. The average age was 9.7+4.3 years. All subjects had dysphagia, 64% had gastroesophageal reflux disease and 70% had constipation. Forty-one percent of patients underwent PEG placement for malnutrition, 47% for aspirations, and 11% for a combination of malnutrition and aspirations. Comparing baseline to T1, weight z-score increased from -5.64+1.59 to -2.87+1.89 (p=0.005), stature z-score from -3.44+2.09 to -2.44+1.98 (p<0.005) and BMI z- score from -5.46+3.05 to -2.18+1.96 (p<0.005).

Moreover, mean MUAC increased from 15.25+3.08 cm to 17.18+3.08 (p=0.138), waist circumference from 47.62+4.75 to 54.8+8.17 (p<0.005), and triceps SFT from 5.74+1.63 to 9.43+2.83 (p=2.27).

**Conclusions**

Neurologically impaired children frequently present with malnutrition and require the placement of a PEG. Our data show that after 1 year from the PEG placement, there is a significant improvement of almost all nutritional parameters, with a probable positive effect on the overall well-being of the individual.

**Ethics Approval**

Study approved by the ethics committee of the Federico II University of Naples.

**References**

1. Scarpato E, Staiano A, Molteni M, Terrone G, Mazzocchi A, Agostoni C. Nutritional assessment and intervention in children with cerebral palsy: a practical approach. Int J Food Sci Nutr. 2017; 68:763-770.
2. Braegger C, Decsi T, Dias JA, Hartman C, Kolacek S, Koletzko B, et al. Practical approach to paediatric enteral nutrition: a comment by the ESPGHAN committee on nutrition. J Pediatr Gastroenterol Nutr. 2010;51:110-22.

## A35. Palivizumab in children with cystic fibrosis: A retrospective study at 10 years

### Savino A^1^, Perna A^1^, Gerbaudo L^1^, Basso S^1^, Folino A^2^, Esposito I^2^, Bignamini E^2^

#### ^1^Department of Pediatrics, University of Turin, Turin; ^2^Department of Pulmonology, Regina Margherita Children Hospital, Città della Salute e della Scienza, Turin

##### **Correspondence:** Savino A (andrea.savino@unito.it)

*Italian Journal of Pediatrics 2023*, **49(Suppl 1):**A35

**Background**

Respiratory Syncytial Virus (RSV) is a leading cause of hospitalization in young infants, both in intensive and non-intensive care, and an important cause of mortality. Nowadays, for children with Cystic Fibrosis (CF), data in the literature on the clinical use of Palivizumab (PVZ) as a prophylaxis against RSV and on the potential benefits or risks in the short and medium term are inconclusive. The aim of this study is to evaluate the PVZ prophylaxis influence in preventing RSV bronchiolitis and in the time and rate of positivisation of microbiological cultures for Staphylococcus Aureus (SA) and Pseudomonas Aeruginosa (PA), in hospital admissions and respiratory function at 10 years.

**Materials and methods**

A retrospective cohort study was approved by ethical Board of Città della Salute e della Scienza. 165 CF children born between 2007 and 2016 were enrolled (84 treated and 81 in the control group) and split into two groups according to the administration or not of two courses of PVZ prophylaxis for RSV at the CF centre of Regina Margherita Children Hospital. The two groups of patients were examined up to 10 years of age and the data on respiratory exacerbation, bacterial colonization of SA and PA, number of hospital admissions and respiratory function values were collected.

**Results**

No RSV-mediated bronchiolitis was found in the group of children who received PVZ prophylaxis. However, these children seem to acquire SA and PA bacterial infections earlier (p<0.05) and experience more respiratory exacerbations and hospital admissions than children without prophylaxis. In particular, in CF children with pancreatic sufficiency that have carried out prophylaxis, infections were contracted earlier (p<0.01) and at 10 years there was a higher chronic colonization compared to the untreated group (p<0.05). Lastly, the increasing number of contracted infections correlates negatively with respiratory function (p<0.01).

**Conclusions**

PVZ prophylaxis is effective in preventing forms of RSV bronchiolitis. The group treated with PVZ shows an earlier bacterial infection with PA and SA and a greater number of hospitalizations, confirming previous studies in the literature. However, further follow-up studies are needed to confirm our data in the long-term and to further clarify the reasons underlying these differences among worldwide centres.

**Ethics approval**

The study was approved by ethical Board of Città della Salute e della Scienza, Torino.

## A36. Congenital hypothyroidism due to TSH receptor mutation: new homozygous mutation in a calabrian family

### Valeria Tallarico, Maria Serena Battigaglia, Giulia Pelaia, Annalisa Giulia Ferlito, Mirella Petrisano, Simona Sestito, Laura Giancotti, Daniela Concolino

#### Pediatric Unit, Magna Graecia University, Catanzaro, Italy

##### **Correspondence:** Valeria Tallarico (valeria.tallarico@libero.it)

*Italian Journal of Pediatrics 2023*, **49(Suppl 1):**A36

**Background**

Primary congenital hypothyroidism (CH) is the most common endocrine disease in children, as well as the most frequent cause of predictable mental retardation which, if not timely recognized and treated, causes irreversible damages. Causes of CH include: thyroid agenesis or dysgenesis, dyshormongenesis, transient forms, syndromic form and genetic causes, such as those concerning TSH receptor, have also been identified as the cause of many forms of CH.

**Material and methods**

We describe three cases of congenital hypothyroidism, in three siblings of related parents (third degree parentage), all identified by newborn screening. The mother presented Hashimoto thyroiditis and hypothyroidism. After the laboratory diagnosis and after the ultrasound examination showing a normal size thyroid gland in the right place, all three patients started hormone replacement therapy. Considering the presence of multiple cases of thyroid disease in the same family, we suspected genetic causes of CH thus performing a Next Generation Sequencing analysis to search for these conditions

**Results**

In all members of the family, the p.M527T mutation in the TSH receptor (*TSHR*) gene (14q31.1) was found. An homozygous condition for this mutation has been identified in the mother and children, while the father was heterozygous for this variant (compared to the other members of the family, he presented only with subclinical hypothyroidism). This variant had never been described in the literature in a homozygous form causing congenital hypothyroidism but was associated, up to now, only with subclinical hypothyroidism due to heterozygous mutation [1].

**Conclusions**

The identification of genetic defects associated with CH is still not routinely carried out today, but it must be proposed when the suspicion of a genetic form is strong. It is important for many reasons: to make an appropriate diagnosis, for the genetic counselling of families with affected members, to identify additional clinical features (such as thyroid cancer risk / nodular thyroid disease), for an adequate follow-up and for the appropriate management of possible complications.

**Consent to publish**

We obtained the written consent for publication from the guardian of the patient.

**References**

1. De Marco G, Agretti P, Camilot M, Teofoli F, Tatò L, Vitti P, et al. Functional studies of new TSH receptor (TSHr) mutations identified in patients affected by hypothyroidism or isolated hyperthyrotrophinaemia. Clin Endocrinol (Oxf). 2009;70:335-8.

## A37. Pathogenesis of gastrointestinal symptoms in Fabry disease: bile acid diarrhea associated with FGF-19 deficiency?

### Valeria Tallarico^1^, Francesca Scionti^2^, Maria Teresa Di Martino^3^, Mariamena Arbitrio^4^, Serena Lavano^1^, Lucy Castaldo^1^, Claudia Palma^1^, Federica Ricca^1^, Simona Sestito^1^, Daniela Concolino^1^, Licia Pensabene^1^

#### ^1^Pediatric Unit, Magna Graecia University, Catanzaro, Italy; ^2^Institute of Research and Biomedical Innovation (IRIB), Italian National Council (CNR), Messina, Italy; ^3^Department of Experimental and Clinical Medicine, Magna Graecia University, Catanzaro, Italy; ^4^Institute of Research and Biomedical Innovation (IRIB), Italian National Council (CNR), Catanzaro, Italy

##### **Correspondence:** Valeria Tallarico (valeria.tallarico@libero.it)

*Italian Journal of Pediatrics 2023*, **49(Suppl 1):**A37

**Background**

Gastrointestinal symptoms (GIS) are often among the earliest presenting events in Fabry disease (FD), an X-linked lysosomal disorder caused by the deficiency of α-galactosidase A [1]. The pathophysiology of the GIS, known to be complex and multifactorial, is still poorly understood. In a previous study, the comparison between FD patients with GIS and FD patients without GIS showed a significant statistical difference in the genotype frequencies of 9 single nucleotide polymorphisms in 4 genes involved in bile acid metabolism (BAs) [2]. BAs can cause diarrhea by a reduced ileal reabsorption or increased synthesis. Bile acid diarrhea (BAD) is a frequent but little-known cause of chronic diarrhea. The deficiency of Fibroblast Growth Factor 19 (FGF-19) has been reported among the underlying mechanisms in BAD [3-4]. The aim of our study was to evaluate the possible association between GIS found in FD patients carrying the previously identified polymorphisms and BAD associated with FGF-19 deficiency.

**Materials and methods**

Serum levels of FGF-19 were assessed in 3 groups: 9 FD patients with GIS (FD GIS) carrying the previously identified polymorphisms, 8 FD patients without GIS (FD no GIS), and 15 healthy subjects (HS). The levels of FGF-19 were measured using the Human FG-19 ELISA Kit (ThermoFisher Scientific, Inc.) according to manual instructions. The reading was performed in duplicate in the Glomax fluorometer (Promega). The FGF-19 expression levels for each sample were interpolated to the standard curve and calculated using the Elisa Analysis software [http://www.elisaanalysis.com]. Statistical analysis was performed with GraphPad Prism 6 using the non-parametric t-test.

**Results**

No significant statistical difference (p <0.05) in the serum levels of FGF-19 was found comparing the groups (FD GIS vs FD no GIS, p=0,5683, FD GIS vs HS, p=0.3689, FD no GIS vs HS, p=0.7965).

**Conclusions**

The preliminary results of this study show no correlation between the serum levels of FGF-19 and the polymorphisms of the genes, involved in the metabolism of BAs, previously found in our FD patients with GIS. However, our results do not exclude a possible correlation with other alterations of the enterohepatic circulation of BAs, not explored in this study. Further studies involving a larger sample size are needed to better understand the role of these polymorphic variants in the pathogenesis of GIS in FD.

**Ethics Approval**

Peripheral blood samples were collected from all participants after appropriate informed consent. The study was approved by the institutional review board in accordance with the Recommendation of the Declaration of Helsinki for biomedical research involving human subjects.

**References**

1. Pensabene L, Sestito S, Nicoletti A, Graziano F, Strisciuglio P, Concolino D. Gastrointestinal symptoms of patients with Fabry disease. Gastroenterol Res Pract. 2016; 2016:9712831.
2. Di Martino MT, Scionti F, Sestito S, Nicoletti A, Arbitrio M, Hiram Guzzi P, et al. Genetic variants associated with gastrointestinal symptoms in Fabry disease. Oncotarget. 2016;7:85895-85904.
3. Walters JR. Bile acid diarrhoea and FGF19: new views on diagnosis, pathogenesis and therapy. Nat Rev Gastroenterol Hepatol. 2014;11:426-34.
4. Johnston IM, Nolan JD, Pattni SS, Appleby RN, Zhang JH, Kennie SLet al. Characterizing factors associated with differences in FGF19 Blood levels and synthesis in patients with primary bile acid diarrhea. Am J Gastroenterol. 2016;11:423-32.

## A38. Organic acidemias and SARS-CoV2 infection: the experience of a reference centre in Campania

### Alessandra Verde^1^, Alessandro Rossi^1^, Simona Fecarotta^1^, Marco Poeta^1^, Francesco Nunziata^1^, Sara Maria Scarano^1^, Federica Giordano^1^, Andrea Lo Vecchio^1^, Alfredo Guarino^1^, Eugenia Bruzzese^1^ and Giancarlo Parenti^1,2^

#### ^1^Department of Translational Medical Sciences, Section of Pediatrics, Federico II University, Naples, Italy; ^2^Telethon Institute of Genetics and Medicine, Pozzuoli, Italy

##### **Correspondence:** Alessandra Verde (verde-alessandra@libero.it)

*Italian Journal of Pediatrics 2023*, **49(Suppl 1):**A38

**Background**

Organic acidaemias are disorders at risk of metabolic decompensation during infections due to a catabolic shift of metabolism. The Centres for Disease Control and Prevention (CDC) suggest that patients affected by inborn errors of metabolism (IEMs) should be considered at high risk of severe illness from COVID-19. There are limited data about SARS-CoV2 infection course or long-term outcome in IEMs. We report the clinical data and management of patients affected by organic acidaemia and COVID-19 in follow up at our centre.

**Materials and methods**

Data about children affected by organic acidaemia and SARS-CoV2 infection were collected both from medical records of Federico II University Covid Paediatric Hub and by telephone interview for patients managed at home through telemedicine, from March 2020 to March 2022. For each patient clinical symptoms, need for hospitalization, oxygen and steroids, therapy changes or underlying disease worsening were recorded.

**Results**

During the study period, 11/81 patients affected by organic acidaemia (M/F 1:1; mean 5.4 ± 4.8 years) acquired SARS-CoV2 infection. Of these, 8 (72%) were symptomatic. The prevalent symptoms were fever (54.4%), cough (36.3%), headache (27.3%), diarrhoea (18.1%), vomiting (9.1%), chest pain (9.1%). 6/11 were managed at home through symptomatic therapy and telemedicine. After an early diet protein intake reduction, 2/11 (1 propionic and 1 methylmalonic acidaemia) were admitted to our hospital for intravenous fluid therapy and described dyspnoea, respectively. No patient developed metabolic decompensation nor required oxygen therapy or intensive care admission. The patient affected by propionic acidaemia showed underlying disease worsening, especially of cardiac and neurological functions, 30 days after acute infection.

**Conclusions**

In our centre experience, individuals with IEMs showed a course of SARS CoV2 infection similar to that reported in general paediatric population. However, these patients have a high risk of metabolic decompensation as during any other infection. Furthermore, careful monitoring of these patients is critical to assess long-term COVID-19 impact. Patients’ management, prevention of metabolic decompensation by appropriate dietary and therapeutic changes, and hospitalization, when necessary, were performed through telemedicine.

**Consent to publish**

Written informed consent for publication of patients information was provided by each patient's legally authorized representative.

